# Substantial Seasonal Contribution of Observed Biogenic Sulfate Particles to Cloud Condensation Nuclei

**DOI:** 10.1038/s41598-018-21590-9

**Published:** 2018-02-19

**Authors:** Kevin J. Sanchez, Chia-Li Chen, Lynn M. Russell, Raghu Betha, Jun Liu, Derek J. Price, Paola Massoli, Luke D. Ziemba, Ewan C. Crosbie, Richard H. Moore, Markus Müller, Sven A. Schiller, Armin Wisthaler, Alex K. Y. Lee, Patricia K. Quinn, Timothy S. Bates, Jack Porter, Thomas G. Bell, Eric S. Saltzman, Robert D. Vaillancourt, Mike J. Behrenfeld

**Affiliations:** 1 Scripps Institution of Oceanography, University of California, San Diego, La Jolla, CA USA; 2 Aerodyne Research Inc, Billerica, MA USA; 3 NASA Langley Research Center, Hampton, VA USA; 4 Science Systems and Applications Inc., Hampton, VA USA; 5 Institute for Ion Physics and Applied Physics, University of Innsbruck, Innsbruck, Austria; 6 The Department of Chemistry, University of Oslo, Oslo, Norway; 7 Department of Civil and Environmental Engineering, National University of Singapore, Singapore, Singapore; 8 Pacific Marine Environmental Laboratory, NOAA, Seattle, WA USA; 9 Joint Institute for the Study of the Atmosphere and Ocean (JISAO), University of Washington, Seattle, WA USA; 10 The Department of Chemistry, University of California, Irvine, Irvine, CA USA; 11 Plymouth Marine Laboratory, Prospect Place, Plymouth, United Kingdom; 12 The Department of Earth System Science, University of California, Irvine, CA USA; 13 The Department of Earth Science, Millersville University, Millersville, PA USA; 14 The Department of Botany and Plant Pathology, Oregon State University, Corvallis, OR USA; 15 Present Address: Cooperative Institute for Research in Environmental Sciences, University of Colorado, Boulder, CO USA

## Abstract

Biogenic sources contribute to cloud condensation nuclei (CCN) in the clean marine atmosphere, but few measurements exist to constrain climate model simulations of their importance. The chemical composition of individual atmospheric aerosol particles showed two types of sulfate-containing particles in clean marine air masses in addition to mass-based Estimated Salt particles. Both types of sulfate particles lack combustion tracers and correlate, for some conditions, to atmospheric or seawater dimethyl sulfide (DMS) concentrations, which means their source was largely biogenic. The first type is identified as New Sulfate because their large sulfate mass fraction (63% sulfate) and association with entrainment conditions means they could have formed by nucleation in the free troposphere. The second type is Added Sulfate particles (38% sulfate), because they are preexisting particles onto which additional sulfate condensed. New Sulfate particles accounted for 31% (7 cm^−3^) and 33% (36 cm^−3^) CCN at 0.1% supersaturation in late-autumn and late-spring, respectively, whereas sea spray provided 55% (13 cm^−3^) in late-autumn but only 4% (4 cm^−3^) in late-spring. Our results show a clear seasonal difference in the marine CCN budget, which illustrates how important phytoplankton-produced DMS emissions are for CCN in the North Atlantic.

## Introduction

Cloud condensation nuclei (CCN) provide the sites on which droplets form, resulting in clouds with radiative properties determined in part by CCN abundance and characteristics. The amount of water that is available to condense is described by the supersaturation, which is often 0.1% for the stratocumulus clouds that cover much of the ocean and reflect a large fraction of incoming sunlight^1–4^. The number and chemical composition of CCN in the marine atmosphere depend on their emission sources and the contributing atmospheric growth processes. The ocean sources of submicron particles are sea spray, which is largely sea salt, and marine biogenic gases that can oxidize and condense, for which dimethyl sulfide (DMS)^5–17^ contributes the most mass. Quantifying these sources for the marine boundary layer aerosol budget provides the framework necessary for predicting how changing ocean conditions will affect marine clouds^18–21^. Model simulations that include parameterizations of marine sources and processes illustrate their effect on the budget of CCN over the remote open ocean. Combining parameterizations of DMS-derived sulfate^22,23^ and sea spray^24^ emission models to simulate CCN contributions showed that DMS-derived sulfate accounted for over 70% of CCN at low wind speeds (<6 m s^−1^) but that sea spray particles contributed more than 80% of CCN at high wind speeds (>12 m s^−1^)^25^. Adding primary marine sea spray particles to a sulfate-only global model increased CCN concentration by less than 20% over most of the North Atlantic, but up to 70% near Greenland (which is frequently influenced by Arctic air masses)^26^. The CCN fraction attributed to primary particles (mostly sea spray) in a global transport model accounted for most CCN at high latitudes but for less than 40% in the mid North Atlantic^27^. These model results reflect substantial uncertainty about what sources are most important for CCN because there are effectively no observations to constrain which model is correct.

The reason for this is that existing measurements provide only limited information about where individual particles come from. Most chemical characterization of aerosol particles over open oceans quantify the mass of different components in particles but not their number^5,28–33^. Because of this, indirect ways to estimate primary marine aerosol contributions have been developed^34–36^. For example, sea spray particles were shown to account for less than 35% of CCN (at 0.1% supersaturation) over most of the North Atlantic and as little as 8% in some regions^36^. However, sampling in clean Arctic air masses had as much as 75% of CCN attributed to sea salt particles^35^. The same number concentrations of sea salt particles accounted for up to 47% of CCN (at 0.1% supersaturation) in clean polar air masses (with few continental sources and low biogenic DMS emissions) but only 8–25% in marine air masses at mid-latitudes (where continental sources contribute more particles and biological DMS sources are larger)^35,37–39^. These indirect approaches have both substantial uncertainty and limited information about particles other than sea spray.

Biogenic sulfate mass has been measured during many open ocean cruises in clean regions^6,9,10,40–43^, with observations in the North Atlantic of 0.06 ± 0.07 μg m^−3^ during winter and 0.45 ± 0.37 μg m^−3^ during summer^44^. In the northeastern Pacific under clean marine conditions^45^, a doubling of the non-sea salt sulfate mass was linked to a 40% increase in CCN concentration. Converting sulfate mass to CCN concentration requires assuming the size and sulfate mass fraction in the particles and does not separate the contributions of nucleation and condensation^46^. There is little evidence of particle nucleation in the boundary layer^6,47–50^, but the conditions in the free troposphere are often more consistent with DMS-derived H_2_SO_4_ nucleation^51,52^. In other words, the cold free troposphere (and clean winter marine boundary layer) has few pre-existing particles and these typically have lower particle surface area than in the boundary layer, making them less likely to compete against nucleation for DMS products^52,53^. This may happen when buoyancy driven transport causes surface air to mix trace gases (such as DMS) throughout the marine boundary layer and sometimes penetrate the mixed layer inversion, transporting trace gases into the free troposphere^54–56^, which is supported by observations^57–64^.

In fact, observations to date indicate that high concentrations of newly formed sulfate particles (<10 nm in diameter) exist in outflow regions of clouds and are important in the nucleation of biogenic DMS products in the free troposphere^50,56,65,66^, where they grow and eventually become entrained in the marine boundary layer^41,65,67–69^ to become an important new source of CCN^69,70^ if rates of entrainment and growth are sufficiently high^70–73^. Global models estimate that the nucleation of DMS products in the free troposphere and entrainment into the boundary layer contribute less than 10% of CCN in the North Atlantic annually^73^. However, direct observations confirming or refuting these modeling results are essentially non-existent. A primary challenge for field verification is the time lag that exists between DMS emission, transport to the free troposphere, oxidation, nucleation, entrainment back down to the boundary layer, and condensational growth to CCN^73–75^. One potential solution to this problem is to use the chemical composition of individual particles in the marine boundary layer to identify CCN sources. Unfortunately, measurements to date have not quantified the sea salt, sulfate, and organic components in individual particles^11,12,76,77^. The current study addresses this issue.

To provide the direct observations of particle composition needed to constrain and evaluate model simulations of CCN, we measured and categorized the chemical compositions of individual marine boundary layer particles and used correlations of particle types to tracers for natural marine (DMS, NaCl, and methane sulfonic acid or MSA) and non-marine (black carbon, radon, hydrocarbon fragment^78^ C_4_H_9_^+^) emission sources to identify measured particle types. Our measurements were conducted during the second Western Atlantic Climate Study (WACS2) and the first and second cruises of the North Atlantic Aerosols and Marine Ecosystems Study (NAAMES1 and NAAMES2). Individual particle mass spectra collected with an Event-Trigger Aerosol-Mass-Spectrometer (ET-AMS) were grouped by k-means clustering into two distinct particle types that contained sulfate and organic components. The lack of markers for fossil fuel hydrocarbons in the mass spectra as well as their higher relative concentration in marine conditions rather than continental conditions indicated the particles were largely from natural marine sources. The centroid of the k-means cluster containing 63% sulfate likely comes from newly formed particles making them “New Sulfate” whereas those with only 38% sulfate are expected to be from existing particles onto which sulfate (or MSA) condensed as “Added Sulfate”. Supporting this attribution, boundary layer inversion strength (a proxy for entrainment rate) was used to distinguish sulfate condensing in the marine boundary layer (Added Sulfate) from sulfate that formed new particles after the lofting of DMS into the free troposphere and entraining back down into the marine boundary layer (New Sulfate). We also constrained sea salt particle number (Estimated Salt) using measured mass composition to get a complete accounting of particle sources for each study. Finally, we calculated the contributions of salt and sulfate particles to CCN using the size and composition of the different types of marine particles.

## Results

**Figure 1:**
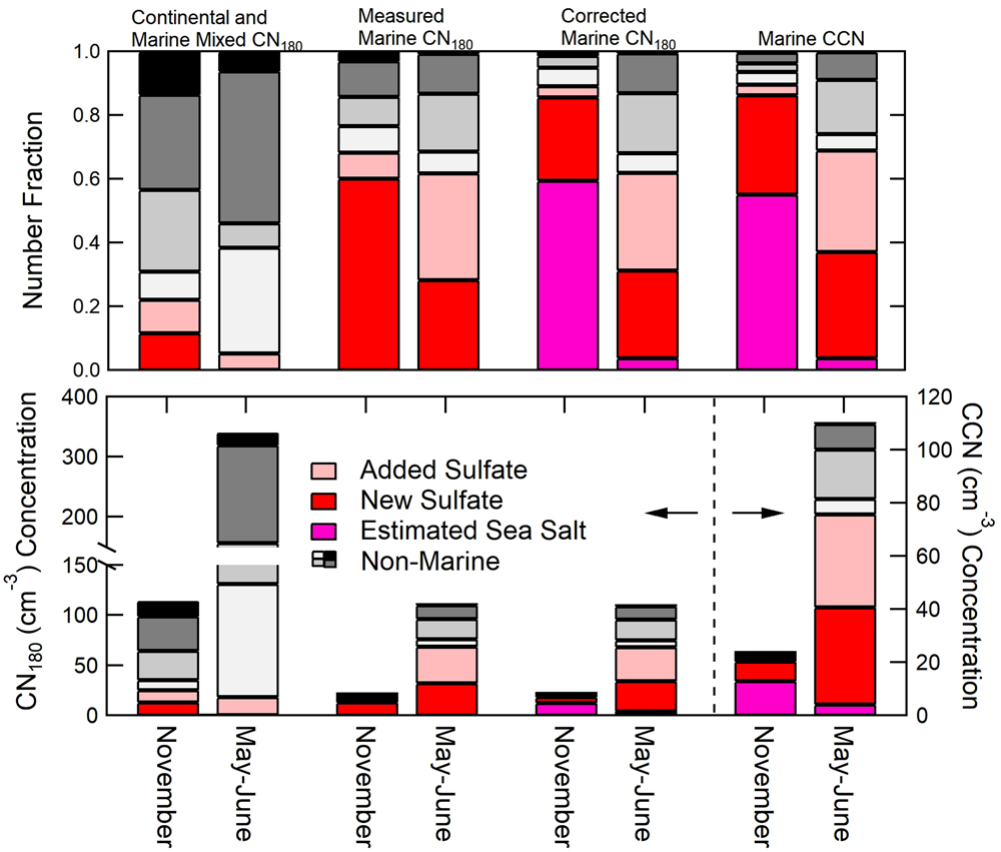
Relative (top) and absolute (bottom) contribution of particles with different chemical compositions measured by ET-AMS in NAAMES1 (November) and NAAMES2 (May-June) for air masses separated for continental (radon greater than 1000 mBq m^−3^) and marine (radon less than 500 mBq m^−3^ and CN concentrations less than 1000 cm^−3^) conditions. Contamination events from the ship stack are excluded. For marine conditions, the measured contributions are also corrected to include Estimated Salt particles (calculated from  Ion Chromatography sodium) and the distributions are integrated to calculate CCN. For the lower panel, the bars to the left of the dotted line correspond to the left axis, and the bars to the right of the dotted line correspond to the right axis. Labels for both plots are found at the top of the figure.

**Figure 2:**
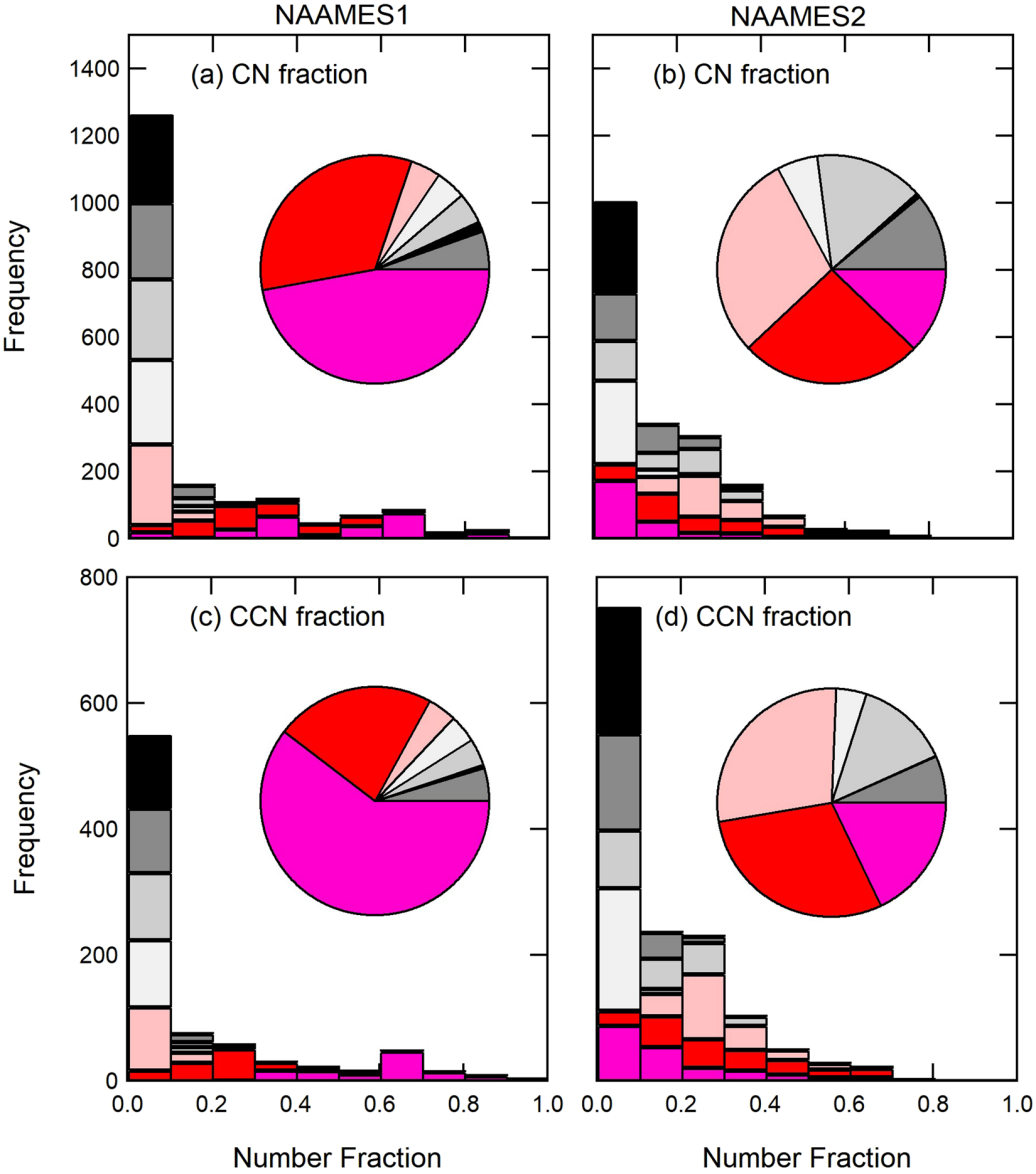
Contributions of seven particle types to (**a**,**b**) CN_180_ and (**c**,**d**) CCN (at 0.1% supersaturation) fraction from clean marine ET-AMS measurements during NAAMES1 and NAAMES2. The histogram frequency represents the number of hours that each particle type accounted for a given number fraction of observed CN_180_ and calculated CCN. WACS2 is excluded because the LS-AMS cut off diameter is 400 nm.

The New Sulfate and Added Sulfate types are the most prevalent type of particles in all of the clean marine air masses that were sampled. For clean marine conditions, the Estimated Salt particles account for 57 ± 22% of Condensation Nuclei (CN) greater than 180 nm in diameter (CN_180_) in November (NAAMES1) but only 4 ± 3% in May-June (NAAMES2), respectively (Figs 1 and 2). The ambient particle concentrations of each measured type are calculated by scaling the measured fraction of each type to the measured particle size-distribution (from combined Differential Mobility Particle Sizer and Aerodynamic Particle Sizer distributions) after subtracting the Estimated Salt distribution.

### Marine Sulfate Sources of Atmospheric Particles

New Sulfate particles accounted for 25 ± 29% and 28 ± 22% of CN_180_ for marine conditions during NAAMES1 and NAAMES2, respectively but less than 15% in continental conditions (Fig. 1). Classification of ambient air as marine or continental is based on the radon concentration, particle concentration, and back trajectories (Supplemental Note). Added Sulfate particles account for 31 ± 20% of particles in NAAMES2 marine conditions but only 3 ± 3% of particles in NAAMES1 (Figs 1 and 2). While the marine measurements satisfy the “clean” criteria for radon, CN concentrations, and back trajectories, contributions from ship emissions and long-range continental transport to the measured sulfate cannot be entirely ruled out. However, the absence of hydrocarbon fragments (m/z 41, 55, 57) in the sulfate particle composition and the lack or negative correlation of the sulfate particle types with black carbon and hydrocarbon-like tracers make the contributions of non-biogenic sources small or unlikely. The continental air masses contain less than 10% Added Sulfate particles, consistent with Added Sulfate particles being of marine origin but still contributing a modicum of particles in polluted conditions. Overall these results provide strong evidence that the three most abundant particle types in the clean marine conditions (New Sulfate, Added Sulfate, and Estimated Salt) are produced by natural ocean sources.

**Figure 3:**
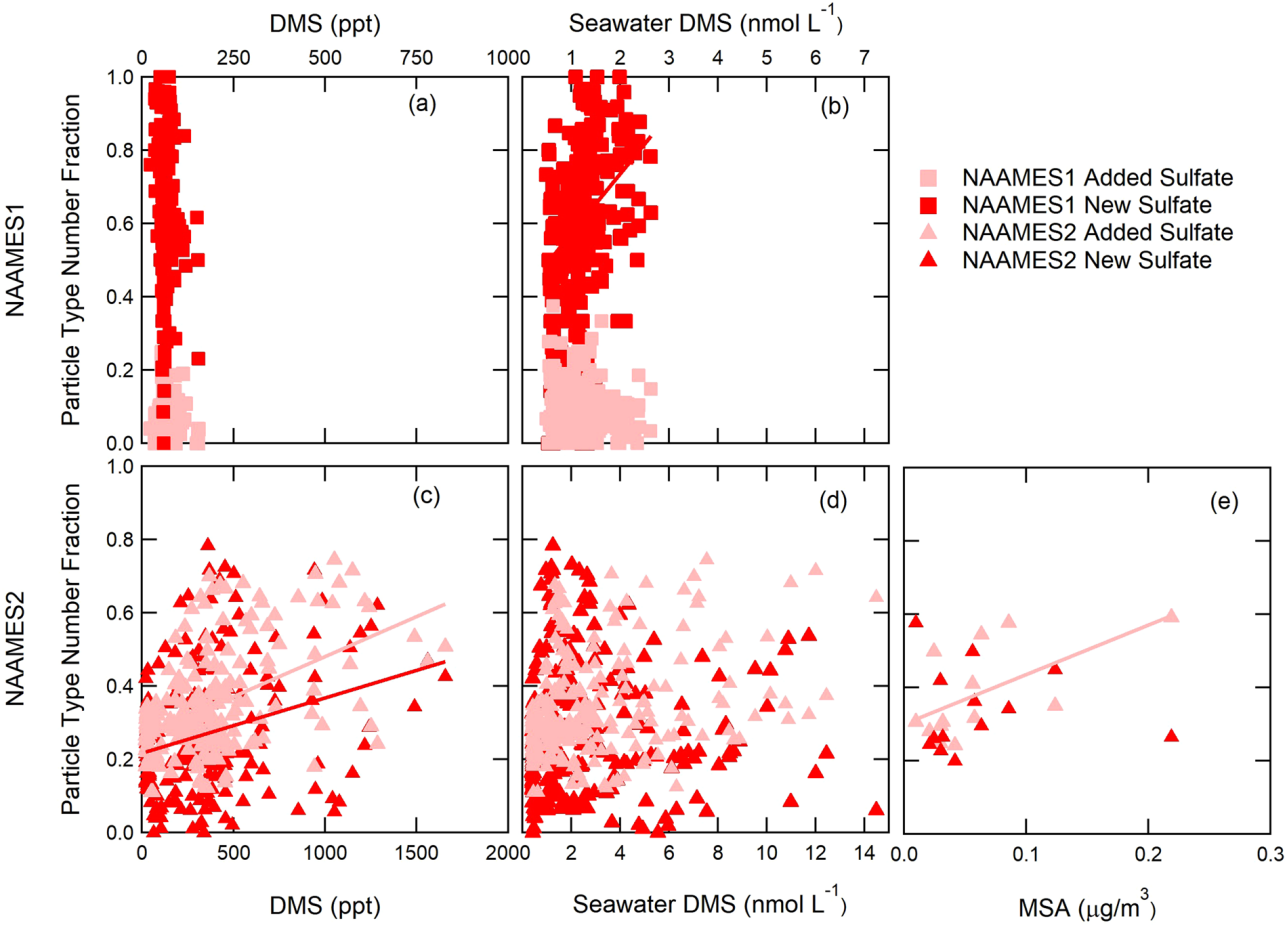
Correlations of NAAMES1 (November) and NAAMES2 (May-June) Added Sulfate and New Sulfate non-refractory particle number fraction to (**a**,**c**) atmospheric DMS, (**b**,**d**) seawater DMS, and (**e**) particle MSA. WACS2 measurements are too few to include. Pearson’s coefficients of correlation are 0.50 and 0.28 for atmospheric DMS to Added Sulfate and New Sulfate particles in NAAMES2, respectively (p < 0.01), 0.37 (p < 0.01) for seawater DMS to NAAMES1 New Sulfate particles, and 0.60 (p = 0.03) for MSA to NAAMES2 Added Sulfate particles. The other relationships show no correlation (r < 0.25).

Further support for the sulfate particles being not only marine but also biogenic is provided by their composition and their correlation to DMS and its products, which are known tracers of phytoplankton emissions. The New Sulfate particle cluster centroid consist of 63% sulfate by mass and the Added Sulfate particle cluster centroid has only 38% sulfate (Supplementary Table S3). The remaining mass is mostly organic (36% for New Sulfate and 58% for Added Sulfate), but the organic fragments were not characteristic of combustion or other continental sources. Added Sulfate particle number fraction is correlated moderately with atmospheric DMS and MSA concentration during NAAMES2 (Supplementary Table S2, Fig. 3), indicating Added Sulfate particles are likely formed from the condensation of DMS products onto existing particles in the boundary layer. (Correlations are defined as weak for |r| > 0.25 and |r| < 0.50, moderate for |r| > = 0.50 and |r| < 0.80, and strong for |r| > = 0.80^79^). The organic (and sea salt) components in Added Sulfate particles (Supplementary Table S3) suggest that the source of the pre-existing smaller particles onto which sulfate is added could include continental, ship, and sea spray emissions, but the organic mass is too small and mixed to identify specific sources.

Interestingly, atmospheric DMS correlated weakly to New Sulfate particle fraction in NAAMES2 (r = 0.28) and did not correlate to any particle types in NAAMES1. This lack of correlation could result from the competition for DMS and its oxidation products with the competing sinks of condensation onto existing particles and vertical transport to the free troposphere. In addition, the long time lag (12–24 hr) between emission, nucleation in the free troposphere, and entrainment to the marine boundary layer means that the time series of sulfate particles may not correlate with DMS even if DMS is the source. For NAAMES1, we find a weak correlation between the New Sulfate particle fraction and seawater DMS (r = 0.37; Fig. 3, Supplementary Table S2), while no correlation was observed during NAAMES2. The observed correlation during NAAMES1 may indicate that the cold temperatures (10.7 ± 5.7 °C), low particle numbers (114 ± 116 cm^−3^), and low particle surface area (17.8 ± 9.6 µm^2^ cm^−3^) allowed new sulfate particles to form from DMS products in the boundary layer without requiring the lofting to the free troposphere that otherwise precludes a correlation in time. We investigated lagged correlations but found nothing significant, likely because the time lag is variable and the transport distances can be large^73^.

The average (standard deviation) sea salt fraction of sulfate (ss-sulfate) mass on the three cruises varied by a factor of nearly 8, with 12 (15%) for WACS2, 52 (28%) for NAAMES1, and 7 (28%) for NAAMES2 (Table 1). NAAMES1 had considerably lower particle concentrations (particle concentrations <50 cm^−3^) and frequent northerly winds as well as some periods with significant ss-sulfate mass fractions. The fraction of ss-sulfate correlates moderately and negatively with particle concentration in NAAMES1 (r = −0.71; Fig. S4), showing that primary marine sea spray particles are a relatively large source in clean Arctic air but a small fraction of higher particle concentrations. The average wind speed was 2.5 m s^−1^ higher during NAAMES1 than NAAMES2, contributing to the higher sea salt mass concentrations in NAAMES1 (Table 1). During NAAMES2 and WACS2, the highest fractions of ss-sulfate mass were during or just after periods of elevated precipitation rates (Fig. S5), likely due to scavenging of particles by precipitation. Scavenging removes all particle types, but sea salt particles are replenished more quickly because sea spray produces particles locally and on shorter time scales than marine biogenic secondary particle production. In summary, these results suggest that the higher ss-sulfate fraction for NAAMES1, relative to NAAMES2, is mostly due the significantly lower biogenic sulfate during NAAMES1.

**Table 1:** Observed Ion Chromography (IC) sea salt and sulfate concentrations and Condensation Nuclei (CN) and Cloud Condensation Nuclei (CCN) concentrations (at 0.1% supersaturation) for clean marine ambient periods during WACS2, NAAMES1, and NAAMES2.

	WACS2	NAAMES1	NAAMES2
180–550 nm^1^			
Sulfate (μg m^−3^)	0.34 ± 0.12	0.07 ± 0.10	0.31 ± 0.14
Sea salt (μg m^−3^)	0.05 ± 0.02	0.25 ± 0.15	0.17 ± 0.14
Sulfate/Sea salt	7.30 ± 3.65	0.25 ± 0.25	3.85 ± 3.66
Sub-1.1 µm^1^			
Sulfate (μg m^−3^)	0.45 ± 0.15	0.14 ± 0.15	0.46 ± 0.22
Sea salt (μg m^−3^)	0.13 ± 0.09	0.99 ± 0.63	0.23 ± 0.20
Sulfate/Sea salt	11.6 ± 16.8	0.39 ± 0.89	3.82 ± 3.35
CN (cm^−3^)	421 ± 127	116 ± 114	423 ± 239
CN_180_ (cm^−3^)^2^	—	22 ± 14	110 ± 81
CCN (cm^−3^)^3^	—	22 ± 12	71 ± 38
Calculated CCN (cm^−3^)^3^	—	26 ± 22	90 ± 54

^1^Only IC measurements that are in clean marine air >75% of the time are included in the mean and standard deviation calculation.

^2^CN greater than 180 nm (CN_180_) are calculated from Differential Mobility Particle Sizer (DMPS) and Aerodynamic Particle Sizer (APS) combined distributions.

^3^Averaged CCN and Calculated CCN are from clean marine periods where CCN, IC, DMPS, and Event-Trigger Aerosol-Mass-Spectrometer (ET-AMS) measurements are all available.

### Entrainment of Particles into the Boundary Layer

**Figure 4:**
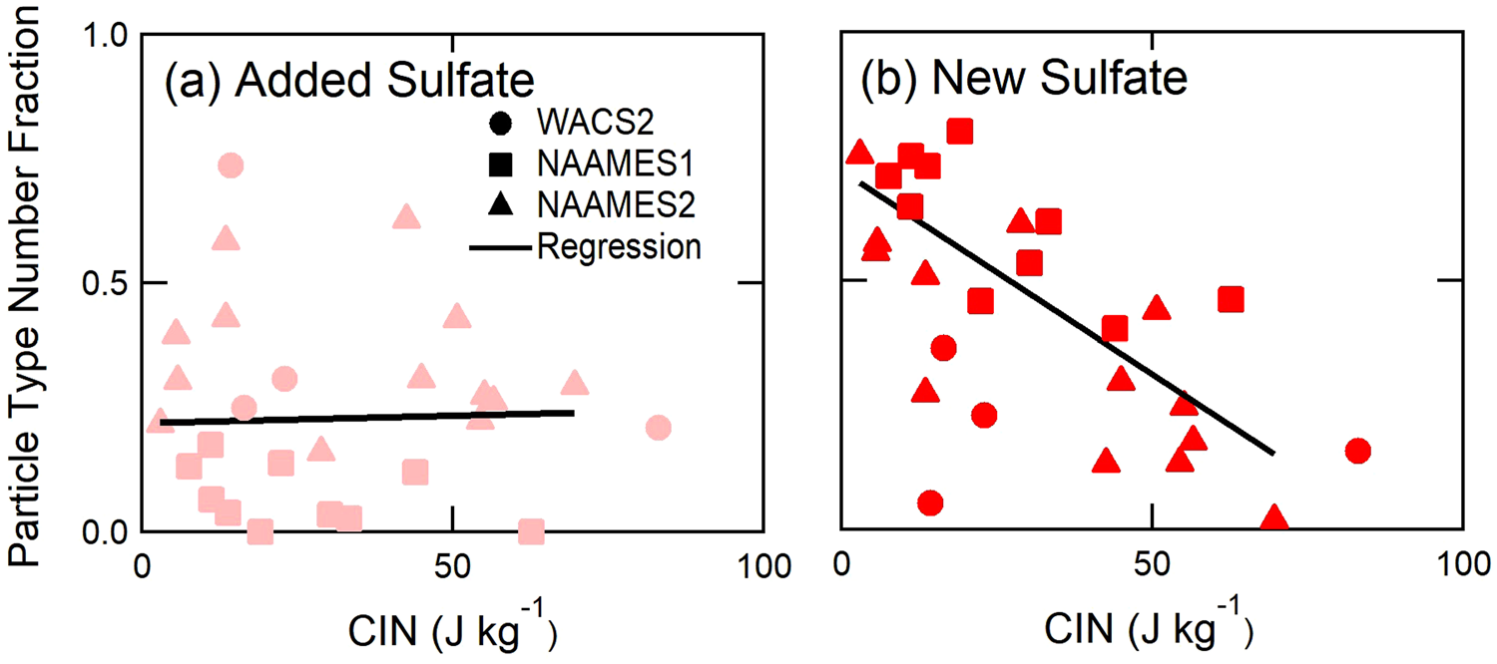
Dependence of WACS2 LS-AMS and NAAMES1 and NAAMES2 ET-AMS non-refractory particle number fractions during clean marine conditions on CIN calculated from radiosonde measurements. Pearson correlation coefficients for NAAMES1 and NAAMES2 for CIN are, (**a**) 0.03 for the Added Sulfate type, and (**b**) −0.76 for the New Sulfate type (N = 24, p < 0.01).

Sources of particles measured at the surface in marine conditions include emissions in the boundary layer itself and transport from the free troposphere. To distinguish particles formed in the boundary layer from those entrained from the free troposphere, the strength of the boundary layer inversion (as indicated by Convective Inhibition or CIN, Supplemental Note) was compared to the number fraction of measured particle types identified by Light-Scattering AMS (LS-AMS) and ET-AMS (Fig. 4). We find that the New Sulfate particle fraction has a moderate and strong negative correlation to inversion strength for NAAMES1 and NAAMES2, respectively, but that the non-marine particle fractions have weak to strong positive correlations. The stronger negative correlation of New Sulfate particle fraction to CIN (r = −0.76) indicates that the New Sulfate number fraction is highest when the boundary layer inversion is weak (indicated by low CIN), providing evidence that New Sulfate particles are frequently entrained from the free troposphere (Fig. 4). Low CIN may also allow increased mixing of DMS up to the free troposphere, providing the source of sulfate^62^.

**Figure 5:**
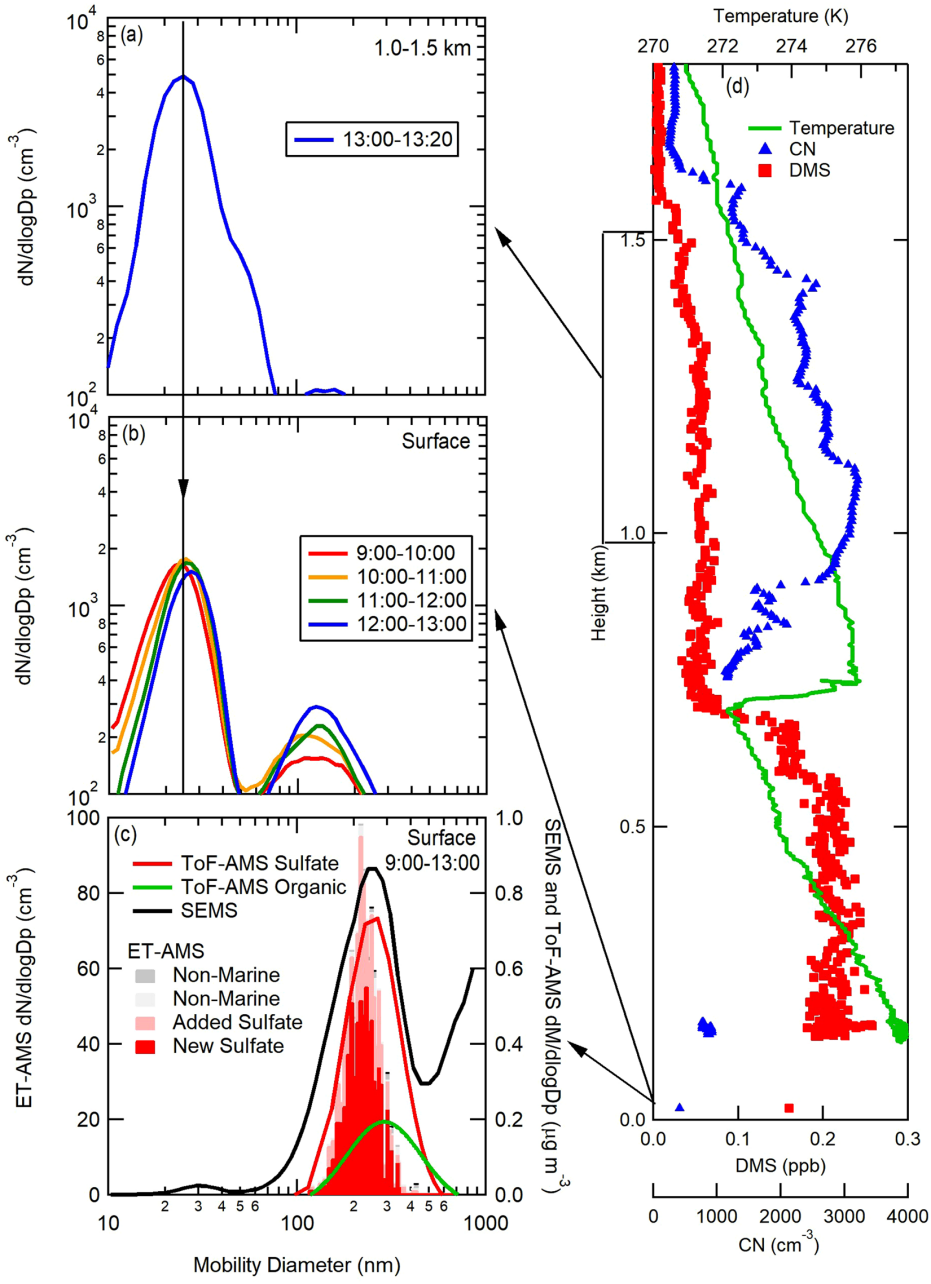
Free troposphere (**a**) and near-surface (**b**,**c**) particle size distributions collected on the NASA C-130 aircraft and *R/V Atlantis* on 20 May 2016 during NAAMES2 (times shown are UTC). The composition of surface measured ET-AMS size distributions are compared to lognormal fits of the sulfate and organic pToF-AMS mass distributions and SEMS mass distributions (**c**). SEMS number distribution was converted to mass using the campaign average density (1.3 g cm^−3^). Vertical profiles (**d**) show temperature, CN from the Condensation Particle Counter (CPC), and DMS concentrations from the Proton-Transfer-Reaction Time-of-Flight Mass Spectrometer (PTR-MS). The two lowest-altitude CN and DMS values in (**d**) were collected on board the R/V *Atlantis*. Particle concentrations have been corrected to cm^−3^ volumes at STP. In-cloud measurements of CN are excluded.

To investigate further these results, we evaluated results from airborne (NASA C-130 aircraft) atmospheric measurements conducted on 20 May 2016 almost directly above the ship (R/V *Atlantis*). These aircraft data indicated surface and free troposphere dry particle size distributions with a mode at approximately 25 nm (Fig. 5a,b). This particle mode is characteristic of recently formed particles since they have a short lifetime and are too small for combustion or other transported primary emissions^80^. Furthermore, we find that these particles are associated with elevated DMS concentrations in the lower free troposphere (Fig. 5d) and give peak concentrations at 25 nm that are almost three times higher at 1–1.5 km than at the surface (measured both on the aircraft and the ship). This finding provides evidence that the source of these particles is the free troposphere and that they were entrained down into the boundary layer (Fig. 5a,b and Supplementary Note). At 25 nm diameter, such particles are not large enough to be active as CCN, but condensational growth from volatile organic compounds and DMS oxidation products can grow them into CCN-sized particles. This process would be consistent with an increase to larger sized particles over time and the observed increase in concentration of the 150 nm mode. For particles with the average chemical composition of New Sulfate (Supplementary Tables S3 and S6), the minimum (or activation) diameter of CCN at 0.1% supersaturation is 156 nm^81,82^. Size distributions of particle Time-of-Flight AMS (pToF-AMS) mass and ET-AMS number show that the accumulation mode consisted largely of sulfate mass and, specifically, of New Sulfate particles (Fig. 5c). This finding provides additional evidence that the entrained particles formed from lofted and oxidized DMS continue to grow to CCN sizes.

**Figure 6:**
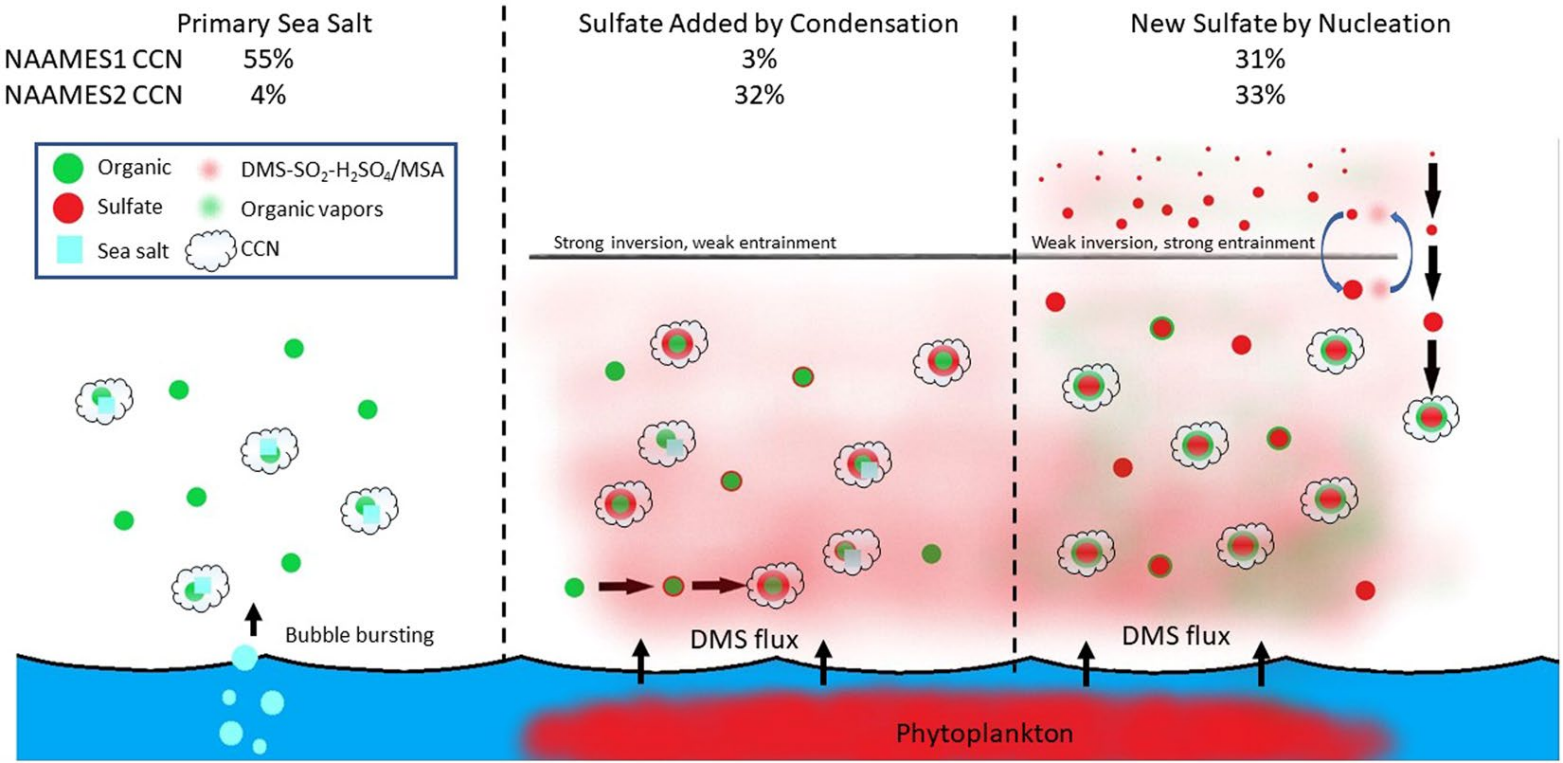
Diagram illustrating the three marine sources of particles, namely primary sea salt (Estimated Salt) from bubble bursting, condensation of DMS oxidation products onto existing particles (Added Sulfate particles) in the boundary layer, and nucleation of DMS oxidation products in the free troposphere before entrainment down to the boundary layer (New Sulfate particles). CCN contributions for NAAMES1 (November 2015) and NAAMES2 (May-June 2016) illustrate the substantial seasonal differences.

Since the New Sulfate particles formed by nucleation of H_2_SO_4_ in the free troposphere are initially smaller than 3 nm^41,83^, condensation of secondary inorganic or organic compounds is needed to grow them to sufficiently large diameters to have lifetimes long enough to be entrained in the boundary layer and to potentially serve as CCN^84,85^. Substantial contributions from organic components would explain why the New Sulfate particles have a significant fraction of organic mass (36%) (Fig. 6 and Supplementary Table S3). Furthermore, we suggest that condensation of secondary organic components onto the New Sulfate particles accounts for the New Sulfate particle organic mass fraction, similar to evidence provided by the diurnal cycle of organic components (Fig. S6).

### CCN Source Contributions

Natural marine particle sources can affect atmospheric radiative properties indirectly by modifying cloud properties^20,21,36^. To quantify these radiative effects accurately, climate models need to be able to correctly simulate natural particle number and CCN budgets. The challenge here is that particle budgets are not uniform across oceans or seasons because marine particle sources are controlled by both physical conditions (wind and sea state) and biological processes (DMS emission) (Fig. 6). In other words, variations in meteorology and ecosystem properties produce different contributions from each of the particle sources. The fraction of those particles that are CCN further depends on particle size and composition-dependent water uptake properties (or hygroscopicity).

We calculated the CCN concentration at 0.1% supersaturation by integrating the number of particles of each type that are larger than the activation diameter of that type (Supplementary Note and Fig. S7). The hygroscopicity parameter (κ) that is needed for calculating the minimum activation diameter of each particle type (Supplementary Table S6) was estimated from the chemical composition as a volume-weighted average of the density and component-specific hygroscopicity of the organic, sulfate, nitrate, and sea salt mass (Supplementary Table S5)^82,86^. We find that the activation diameters at 0.1% supersaturation for Added Sulfate, New Sulfate, and Estimated Salt particles have similar values, ranging from 130 to 183 nm (Supplementary Table S6). For CCN spectra collected at 0.1% intervals, the activation diameter differs by 50 to 68 nm between 0.1% and 0.2%^36,87^. What this means is that all three particle types activate in the same supersaturation bin, thus giving a sharp step change in spectra despite their differing chemical compositions. Our calculated CCN concentrations are on average within 16% and 22% of measured CCN concentrations for NAAMES1 and NAAMES2, respectively (Figs 1 and 2).

From a seasonal perspective, we find that Estimated Salt particles account for 57 ± 22% of CCN in November (NAAMES1), but only 4 ± 3% during the clean marine conditions sampled in May-June (NAAMES2). Since the Estimated Salt particles account for a smaller mass fraction at smaller diameters (<100 nm)^6^, these particles would account for fewer CCN at supersaturations higher than 0.1%^34^. Excluding the organic component from the Estimated Salt type had no effect on the Estimated Salt CCN concentration because the increase in particle hygroscopicity offsets the decrease in particle size. The significance of these findings is that clouds with higher updraft velocities (such as cumulus) would have larger fractions of New Sulfate and Added Sulfate.

New Sulfate particles accounted for an average of 33 ± 24% of CCN (at 0.1% supersaturation) during NAAMES2 clean marine sampling and for an even greater percentage (55 ± 19%) at times when the boundary layer inversion was weak. For NAAMES1, the New Sulfate particles accounted for 31 ± 37% of CCN for all clean marine air masses and only slightly more (36 ± 24%) during weak boundary layer inversions. Since the New Sulfate particles are small when formed by nucleation, they could frequently represent a larger fraction of particles smaller than the ET-AMS cutoff diameter (145–180 nm, Supplementary Fig. S2 and Table S4). What these findings suggest is that New Sulfate particles would represent more than 31% and 33% of CCN at supersaturations higher than 0.1% for NAAMES1 and NAAMES2, respectively. Finally, we found that Added Sulfate particles account for 32 ± 20% of CCN (Figs 1 and 2) and, at higher atmospheric DMS concentrations (>500 ppt), they account for the majority of CCN for NAAMES2. This same class of particles only accounted for 3 ± 3% of CCN during NAAMES1.

## Discussion

One important finding of this study was that sea spray particles are a large fraction (>50%) of a very small number (25 cm^−3^) of natural marine CCN at 0.1% supersaturation in November (NAAMES1), largely because the low phytoplankton productivity emits little DMS and consequently few New or Added Sulfate particles. In contrast, the phytoplankton bloom conditions of May-June (NAAMES2) in the North Atlantic provide three times more CCN (90 cm^−3^), of which less than 5% are from sea spray (Fig. 1, Table 1). Nearly one-third of these CCN (32%) in May-June are produced from DMS oxidation products that nucleate New Sulfate particles, and another third (31%) is from Added Sulfate on pre-existing particles. These substantial seasonal differences in number concentrations provide constraints for models to test their process parameterizations.

An interesting consequence of measuring specific marine particle types is the new evidence for nucleation of DMS products in the free troposphere that is provided by the negative correlation of New Sulfate particles to weak boundary layer inversions (low CIN). This result provides substantial evidence for particle nucleation occurring after lofting DMS to the free troposphere. Going further on this point, the weaker correlation to seawater DMS in NAAMES1 suggests that the colder temperatures and lower particle concentrations of November may have supported nucleation of DMS-derived H_2_SO_4_ in the boundary layer rather than the free troposphere. This possibility could also explain the small number (3%) of Added Sulfate particles because New Sulfate formation is a faster sink of DMS oxidation products if transport to the free troposphere is not required. If this interpretation is further substantiated by future studies, it provides strong evidence for the biogenic contribution to cloud radiative properties.

Based on these results, we can also consider what would happen if DMS emissions from phytoplankton were decreased. Removing the DMS oxidation products that nucleate would eliminate all New Sulfate particles to lower CCN by more than 30% in both November and May-June, with the expectation that the organic components would redistribute and condense onto existing particles without increasing CCN. We find that removing the sulfate mass from the Added Sulfate particles (using densities in Supplementary Table S5) results in 60% fewer Added Sulfate CCN at 0.1% supersaturation, indicating that without the condensation of DMS oxidation products there would be 19% fewer CCN in May-June (NAAMES2) but little change (2%) in November (NAAMES1). The summed effects of removing DMS contributions to both Added Sulfate and New Sulfate particles eliminates an average of 9 cm^−3^ CCN (33%) in November (NAAMES1) and 47 cm^−3^ CCN (52%) in May-June (NAAMES2). Alternatively, if we double the biogenic sulfate in November as a hypothetical response to warmer temperatures and more productive phytoplankton, CCN would increase by 33%.

These DMS-driven changes in CCN concentration are expected to influence cloud droplet concentrations, but cloud processes can buffer their impact on cloud properties^88^. For example, increased CCN concentrations could reduce precipitation, which could increase or decrease cloud lifetime^89,90^. But if we consider only the initial changes in cloud drop number concentration associated with the CCN differences for an idealized cloud (100 m thick, 283 K, 0.3 g/kg liquid water at cloud top) that activates all CCN at 0.1%, then the increase in albedo from adding 50% more DMS-related CCN is 13% in November but eliminating biogenic DMS decreases the albedo by 52% in May-June. These results provide the most direct evidence to date of the proposed link between greater DMS emissions and more CCN^20,21^. Moreover the results provide season-specific constraints on the magnitude of its impact on CCN that could be used to evaluate the fidelity of global simulations of CCN from DMS and sea spray^25–27,73,91–96^. Toward this end, simulations that track CCN from Sea Spray in addition to Added Sulfate and New Sulfate as separate particle types would enable quantitative evaluation of the new particle formation rates used.

## Methods

WACS2, NAAMES1, and NAAMES2 included comprehensive chemical and physical characterization of atmospheric aerosol particles. WACS2 sampled in the northwestern Atlantic aboard the R/V *Knorr* from 20 May to 5 June 2014 between 33°N and 42°N and between 61°W and 71°W. NAAMES1 and NAAMES2 sampled in the North Atlantic from 6 November to 1 December 2015 and 11 May to 5 June 2016, respectively. During NAAMES1 the R/V *Atlantis* transited approximately to the northeast until 55°N at 40°W then headed southward to 40.5°N, 40°W. For NAAMES2, the R/V *Atlantis* followed a similar track, from 56.5°N, 47°W to 44°N, 43°W.

### Aerosol Particle Measurements

On all three cruises, ambient particles were collected with a temperature-controlled isokinetic inlet at approximately 18 m above sea level and dried in diffusion driers before being transported to the instruments reported here. Supermicron particles were removed by a 1.0 µm sharp cut cyclone (SCC 2.229, BGI Inc. US). A Condensation Particle Counter (CPC 3010, TSI Inc., St. Paul, MN) was used to identify contamination from ship exhaust. Submicron particles were analyzed with a high-resolution time-of-flight aerosol mass spectrometer (AMS, Aerodyne Research Inc., Billerica, MA)^97^ that measures non-refractory inorganic (sulfate, ammonium, nitrate, chloride) and organic components.

During WACS2, the AMS included LS-AMS mode to analyze the composition of individual particles^98^ for particles with mobility diameter greater than 400 nm^99^. During NAAMES1 and NAAMES2, the AMS included the ET-AMS mode, which extracted mass spectra for individual particles that had ion signals exceeding pre-set thresholds for three m/z regions^100^. NAAMES1 ET-AMS thresholds were typically set to 4.5 ions/extraction at m/z 55–79, 6 ions/extraction at m/z 48–150, and 4 ions/extraction at m/z 43 or 2.5 ions/extraction at m/z 48. NAAMES2 used the same m/z regions (excluding m/z 48) but with trigger levels of 8, 9, and 3.5 ions/extraction. Higher trigger levels were chosen for NAAMES2 to account for the higher single ion baseline and air beam intensity associated with the higher particle concentrations. The thresholds were determined using particle-free air. The LS-AMS and ET-AMS measurements were processed by Sparrow software version 1.04E and Tofware version 2.5.3.b (TOFWERK and Aerodyne Research, Inc.). The pre-processed data were clustered using the clustering input preparation panel (CIPP) v1.2 and the clustering analysis panel (CAP) v1.2 (developed by A. Lee, National University of Singapore, and M. Willis, University of Toronto), which applies a k-means clustering algorithm to the mass spectra of the particles^101,102^. Only particles with mass spectra signal-to-noise ratio of greater than 5 were used in the clustering analysis (Table S4). WACS2 also included a second AMS with a soot-particle module (SP-AMS, Aerodyne Research Inc., Billerica, MA) operated in standard AMS mode (with tungsten vaporizer on and laser vaporizer off) to evaluate the effect of volatility temperature on salt detection. All diameters from the ET-AMS, LS-AMS and pToF-AMS were converted to mobility diameter using a shape factor of 1 and campaign average particle type densities (Fig. S2).

Mass spectra of ambient single particles with diameters greater than 180 nm from the Event-Trigger AMS (ET-AMS) during NAAMES1 (in November 2015) and NAAMES2 (in May and June 2016) were grouped by k-means clustering to identify three types of spectra, all of which are similar to spectra identified by AMS ensemble (non-single-particle) mode measurements: hydrocarbon-like organic aerosols (HOA), oxygenated organic aerosols (OOA), and sulfate-containing particles^99,102–106^ (Fig. 1, Supplementary Note, Tables S2, S3 and S6, and Figs S1 and S2). WACS2 particle type fractions from the Light-Scattering Aerosol-Mass-Spectrometer (LS-AMS) are shown separately (Fig. S3) for particles greater than 400 nm diameter. The collection efficiency of the AMS sea salt (CE_SS_) is calculated as 3.26*Na^+^, which account for the mass of sodium chloride, magnesium sulfate and other inorganic salts present in seawater, where Na^+^ is the sodium concentration from Ion Chromatography (IC) measurements. Non-marine particles are described in the Supplementary Note. The refractory sea salt particles missed by the HR-AMS are calculated as Estimated Salt particle number concentration from the ET-AMS number size distribution scaled to the collection-efficiency-corrected AMS sea salt mass (Figs 1, 2 and Supplemental Note). Sea salt (ss) sulfate is calculated by dividing the amount of sulfate associated with sea salt (7.7% of sea salt mass)^107^ from the total sulfate mass measured by IC (Table 1).

On all three cruises, ambient particles were collected on pre-scanned 37 mm Teflon filters (Pall Inc., 1 µm pore size) for 4 to 24 hr for Fourier transform infrared (FTIR) spectroscopy (Tensor 27 spectrometer, Bruker, Billerica, MA). The particles were dried in diffusion driers and passed through either a 1 µm sharp cut cyclone (SCC 2.229 BGI Inc., U.S.) or a 1.1 µm cut Berner impactor. The FTIR spectrum from each filter was baselined and integrated at specific peak locations to determine the peak areas of the organic functional groups using an automated algorithm^108–110^. Ambient particles were also collected on Millipore Fluoropore filters with a 1.1 µm cut Berner impactor for extraction and IC for sodium, chloride, sulfate, nitrate, and ammonium mass^40^.

On all three cruises, a Differential Mobility Particle Sizer (DMPS, University of Vienna^111^) was used to measure the number size distribution of dry submicron (0.02–0.8 µm diameter) ambient particles^112^. Supermicron particle size distributions were measured using an Aerodynamic Particle Sizer (APS 3321,TSI Inc., 0.5 < Dp < 20 µm). Radon was measured with a dual-flow-loop two-filter 103 radon detector^113^. During NAAMES1 and NAAMES2, a Scanning Electrical Mobility Sizer (SEMS, Model 138, 2002, BMI, Hayward, CA) measured particle size distributions and a Single-Particle Soot Photometers (SP2, DMT, Boulder, CO) measured refractory black carbon number and mass concentration^114^. Continuous DMS measurements were made by atmospheric pressure chemical ionization mass spectrometers^115,116^. One instrument was dedicated to air measurements and the other analyzed gas that had been equilibrated with seawater. During NAAMES1 and NAAMES2, a Cloud Condensation Nuclei Counter (CCNC, DMT, Boulder, CO) measured ambient CCN concentrations at 0.1% supersaturation^117^.

### Aircraft and Balloon Microphysical and Meteorological Measurements

The NASA C-130 aircraft collected aerosol particle measurements between 100 m and 3000 m near the location of the R/V *Atlantis* during NAAMES1 and NAAMES2. A Scanning Mobility Particle Sizer (SMPS, TSI Inc., Shoreview, MN) measured ambient aerosol number size distribution (0.01 to 0.3 µm diameter) at multiple heights. A Condensation Particle Counter (CPC 3772, TSI Inc., St. Paul, MN) measured the particle concentration. Both SMPS and CPC measurements are reported at standard temperature and pressure (T = 0 °C, P = 1013 mb). A Proton-Transfer-Reaction Time-of-Flight Mass Spectrometer (PTR- MS)^118^ was used to measure volatile organic compounds including DMS.

Radiosondes (iMet-1) were launched twice daily typically between 1000 and 1200 UTC and between 1800 and 2000 UTC for NAAMES1, NAAMES2 and WACS2. The radiosondes directly measure temperature, pressure, and relative humidity, which were used to calculate the inversion strength at the top of the marine boundary layer (Supplementary Note)^119,120^. The inversion strength is useful because it has been shown to be correlated negatively to the entrainment rate^121–123^.

### Data Availability

The datasets generated during and analyzed for the WACS2 study are available in the UCSD Library Digital Collection repository, 10.6075/J0M61HFH and PMEL repository, https://saga.pmel.noaa.gov/data/PrePlot.php?cruise=WACS2014.

The datasets generated during and analyzed for the NAAMES1 and NAAMES2 studies are available in the UCSD Library Digital Collection repository, 10.6075/J04T6GJ6, in the PMEL repository, https://saga.pmel.noaa.gov/data/download.php?cruise=NAAMES1 and https://saga.pmel.noaa.gov/data/download.php?cruise=NAAMES2, and the NASA repository, https://www-air.larc.nasa.gov/missions/naames/index.html.

## Electronic supplementary material

Supplementary Information
