# Supplementary material for: Substantial Seasonal Contribution of Observed Biogenic Sulfate Particles to Cloud Condensation Nuclei
Source: Sci Rep. 2018 Feb 19;8:3235. doi: 10.1038/s41598-018-21590-9 (PMC5818515; doi:10.1038/s41598-018-21590-9)
Supplement: Supplementary file 1 — Supplementary Information [file 41598_2018_21590_MOESM1_ESM.docx]

Supplementary Information for

**Substantial Seasonal Contribution of Observed Biogenic Sulfate Particles to Cloud Condensation Nuclei**

Kevin J. Sanchez^1^, Chia-Li Chen^1^, Lynn M. Russell^1*^, Raghu Betha^1^, Jun Liu^1^, Derek J. Price^1+^, Paola Massoli^2^, Luke D. Ziemba^3^, Ewan C. Crosbie^3,4^, Richard H. Moore^3^, Markus Müller^5^, Sven A. Schiller^5^, Armin Wisthaler^5,6^, Alex K. Y. Lee^7^, Patricia K. Quinn^8^, Timothy S. Bates^8,9^, Jack Porter^10^, Thomas G. Bell^11,12^, Eric S. Saltzman^12^, Robert D. Vaillancourt^13^, Mike J. Behrenfeld^14^

1 Scripps Institution of Oceanography, University of California, San Diego, La Jolla, CA, USA

2 Aerodyne Research Inc., Billerica, MA, USA

3 NASA Langley Research Center, Hampton, VA, USA

4 Science Systems and Applications Inc., Hampton, VA

5 Institute for Ion Physics and Applied Physics, University of Innsbruck, Innsbruck, Austria

6 The Department of Chemistry, University of Oslo, Oslo, Norway

7 Department of Civil and Environmental Engineering, National University of Singapore, Singapore

8 Pacific Marine Environmental Laboratory, NOAA, Seattle, WA, USA

9 Joint Institute for the Study of the Atmosphere and Ocean (JISAO), University of Washington, Seattle, WA USA

10 The Department of Chemistry, University of California, Irvine, CA, USA

11 Plymouth Marine Laboratory, Prospect Place, Plymouth, United Kingdom

12 The Department of Earth System Science, University of California, Irvine, CA, USA

13 The Department of Earth Science, Millersville University of Pennsylvania, Millersville, PA

14 The Department of Botany and Plant Pathology, Oregon State University, Corvallis, OR, USA

^+^ Now at the Cooperative Institute for Research in Environmental Sciences, University of Colorado, Boulder, CO USA

*Correspondence should be addressed to (lmrussell@ucsd.edu)

**Supplementary Note**

This note provides additional details on the particle type clustering criteria, the Non-Marine particle types, the sea salt mass correction, the Estimated Salt correction, the HR-AMS vaporizer temperature effects, the hygroscopicity and CCN calculations, and the inversion strength calculations.

*Particle Type Clustering Criteria*

Supplementary Table S4 summarizes the single particle measurements from WACS2, NAAMES1, and NAAMES2. The individual particle mass spectra were grouped using k-means clustering into 7-10 clusters, which were then compared and similar clusters were identified and combined. NAAMES1 and NAAMES2 ET-AMS ambient particles included one HOA, three OOA and two sulfate clusters; WACS2 LS-AMS types were the same but had no HOA type (Supplementary Figure S1). The sulfate particle types have peaks at m/z 48 (SO^+^), m/z 64 (SO_2_^+^), m/z 80 (SO_3_^+^), m/z 81 (HSO_3_^+^), m/z 96 (SO_4_^+^), and m/z 98 (H_2_SO_4_^+^). The HOA (C_x_H_y_) cluster has peaks in the alkane series C_x_H_2y-1_^+^ and C_x_H_2y+1_^+^ with high m/z 41 (C_3_H_5_^+^), m/z 43 (C_3_H_7_^+^), m/z 55 (C_4_H_7_^+^), and m/z 57 (C_4_H_9_^+^). The OOA particles contain mostly m/z 44 (CO_2_^+^) and some m/z 43 (C_2_H_3_O^+^). The OOA and HOA cluster spectra are similar (with cosine similarity greater than 0.8) to LS-AMS spectra in WACS marine aerosol^1^. The HOA cluster was similar to primary emissions from fossil fuel (FF) combustion^2,3^. The three OOA clusters include one with mostly less oxidized (LO) organic components, one with more oxidized (MO) organic components, and a third considered to be “mixed continental” (MC) because it contains both sulfate and organic mass and correlates moderately with radon during NAAMES2 (r = 0.55). The MO, LO, FF and MC particle number concentrations correlate with one or more continental tracers and are typically higher fractions of particle number in continental air masses and therefore are identified as Non-Marine particles (Figure 1, Supplementary Table S2 and Figure S3).

The FF, LO and MO organic and two sulfate particle types were similar to previously measured mass spectra (Supplementary Table S2), with cosine similarity ranging from 0.6 to 0.8. The main differences among the sulfate particle mass spectra were the relative amounts of sulfate and organic peaks, which was likely caused by differences in fragmentation associated with matrix effects and vaporizer inhomogeneities rather than differences in composition. Consequently, the sulfate spectra were grouped into two types, one that contained mostly sulfate mass and one that contained mostly organic mass (Supplementary Table S3). The time series of the two sulfate clusters were correlated weakly (r < 0.4).

*Non-Marine Particle Types*

The Non-Marine particle types identified from the ET-AMS and LS-AMS mass spectra are likely from different primary and secondary emission contributions^4-6^. Radon is a continental tracer (the main source is soil) that has been shown to be correlated to other emissions from land-based sources^7^. We use the level of radon and CN to separate marine (radon < 500 mBq m^-3^, CN < 1000 cm^-3^) and continental (radon > 1000 mBq m^-3^, CN > 1000 cm^-3^) air masses for the three campaigns (Figure 1, Supplementary Figure S3)^8^. Marine conditions also had back trajectories that spent more than 75% of the preceding 5 days over the ocean. Continental measurements are not shown for WACS2, because very few measurements had high radon concentrations. The clean marine periods with IC measurements that are used in Table 1, Figure 1, and Supplementary Figure S3, S4 and S5 are 17:35 21 May 2014 – 22:04 21 May 2014, 23:24 28 May 2014 – 10:41 31 May 2014, 22:18 11 November 2015 – 08:00 17 November 2015, 19:00 18 November 2015 – 08:00 20 November 2015, 19:36 22 November 2015 – 06:41 24 November 2015, 10:30 15 May 2016 – 7:55 17 May 2015, and 18:17 24 May 2016 – 09:10 01 June 2015.

The LO particle type contributes the largest fraction of particle number to continental air masses (Figure 1) and correlates moderately with radon in both NAAMES1 (r = 0.40) and NAAMES2 (r = 0.49). The FF particle type is also more abundant in continental air than marine air for both NAAMES1 and NAAMES2. This FF particle type correlates moderately to a previously identified AMS marker fragment associated with fossil fuel combustion C_4_H_9_ (r = 0.70)^9^ and to black carbon number concentration (r = 0.54)^10^, suggesting these particles come from anthropogenic fossil fuel combustion sources that could include ships (Supplementary Figure S8). The FF particles were also measured at high concentrations when the ship stack was upwind of the sampling line (time periods with ship stack contamination are excluded from both the continental and clean marine periods). The FF particle type was not identified in the WACS2 LS-AMS measurements, likely because there were no periods with continentally-influenced air masses or with ship stack emissions. The MC particle type also likely has a continental source because of the moderate correlation to radon in NAAMES2 (r = 0.55, Supplementary Table S2) and its higher concentration during continental conditions in NAAMES2 (Figure 1). The MC particle type contributes a much smaller fraction of particles during continental periods in NAAMES1 than NAAMES2, in line with the generally lower concentrations and cleaner conditions during NAAMES1 in both continental air (1070 ± 720) and marine air (116 ± 114). Note that there is still approximately 10 times higher number concentration of MC particles in continental conditions than in marine conditions.

The MO particle type is not consistently higher or lower in continental air masses than in marine air masses, suggesting a particle type that forms in both marine and continental air masses. The MO particle type has almost the same ET-AMS chemical composition as the LO particle type (Supplementary Table S3) but a higher fraction of oxidized organic fragments (m/z 44, CO_2_; m/z 29, CHO) than the LO type. This suggests that the MO particle type consists of marine or continental particles that have had a longer residence time in the atmosphere and have accumulated a substantial amount of photochemically-produced secondary organic aerosol. This long residence time in the atmosphere would also explain the lack of association with either marine or continental emission tracers. In NAAMES2, the MO and LO types both correlate weakly to an AMS biomass burning marker fragment (m/z 60, C_2_H_4_O_2_), suggesting the transported particles may include contributions from wildfire emissions^11^ with longer (MO) or shorter (LO) residence times. The diurnal variation of the ratio of MO to LO particles also provides evidence of condensation of secondary organic compounds onto particles in the marine boundary layer (Supplementary Figure S6). The greater MO particle fraction in the afternoon suggests secondary organics are condensing onto LO particle types. In effect, MO particles cannot be identified with specific emissions because their source signatures are largely atmospheric rather than marine or continental. The ratio of the MO to LO particle concentration has a peak in late afternoon, suggesting that LO particles are being oxidized to form more MO particles (Supplementary Figure S6).

MO, MC, LO and FF particles account for 1%, 1%, 1% and 0%, respectively, for NAAMES1 and 11%, 4%, 6%, and 0%, respectively, for NAAMES2. MC and FF particles have small contributions to CCN because they are from continental and anthropogenic sources that are largely excluded during clean marine conditions. The small but consistent fraction of the MC and LO particles that account for up to 11% of CCN are consistent with contributions from non-marine sources observed in other clean marine conditions^12,13^.

*Sea Salt Mass Calculation*

The sea salt in all particle types is estimated using the sum of chloride peaks in their mass spectra. WACS2, NAAMES1, and NAAMES2 included comprehensive chemical and physical characterization of particles from SeaSweep^14^ which generates particles on the sea surface, free of influence from or processing in ambient air^14^. The sea salt type measured from SeaSweep (Supplementary Figure S1) for NAAMES1 has a lower sulfate/chloride ratio than expected (0.16)^15^ for seawater. The sulfate ion fraction was corrected from 1% to 5% for consistency with the expected sulfate/chloride ratio of 0.16 (Supplementary Table S3). The chloride and corrected sulfate ion fraction is divided by 0.627, the mass fraction of chloride and sulfate in seawater^15^. During NAAMES1 SeaSweep measurements, the ET-AMS m/z 43 region, used for ambient measurements, was replaced by m/z 23, 35 or 58 to measure sea salt particles. The sea salt type was not observed in NAAMES2 because the ET-AMS did not select for m/z 23, 35 or 58. NAAMES1 sea salt composition was used for NAAMES2 sea salt because the campaigns contain similar sea salt mass concentrations (Table 1).

SeaSweep particles were also collected on Millipore Fluoropore filters with a 1.1 µm cut Berner impactor for extraction and IC for sodium^16^. The IC measurements of Na^+^ collected on PM1 filters were used to calculate the mass of sea salt as 3.26*Na^+^ (based on calculations from Quinn et al.^17^ and the ratio of sea salt ions in seawater^15^). The HR-AMS sea salt collection efficiency (CE_ss_)^1^ was calculated as the ratio of the summed salt masses (Cl­^+^, HCl^+^, NaCl^+^, Na_2_Cl^+^, KCl^+^, MgCl^+^, ^37^Cl^+^, H^37^Cl^+^, Na^37^Cl^+^, Na_2_ ^37^Cl^+^, K^37^Cl^+^, ^41^KCl^+^, ^41^K^37^Cl^+^ and Mg^37^Cl^+^) to the IC sea salt mass . The Berner impactor stages for the 180 nm – 550 nm diameter range are used because salt below 180 nm was below detection and HR-AMS does not measure 50% of particles above 700 nm (Supplementary Figure S2).

*Estimated Salt Correction*

The Estimated Salt particle concentration is determined by apportioning the sea salt mass calculated from the HR-AMS salt mass after correction by CE_ss_ to the ET number distribution as follows:

$N_{ETsalt,j}=(1+X_{sweep})\frac{M_{HRSS}}{{CE}_{SS}}\frac{N_{ET,j}}{{\sum M}_{ET,j}}$ (1)

$N_{ET,j}$ is the number concentration of the campaign average ET-AMS particle number concentration of all ET-AMS types at particle diameter j. $M_{ET,j}$ is the ET-AMS particle mass calculated from $N_{ET,j}$ using a density of 1.73 g cm^-3^ (from 70% sodium chloride, 10% sulfate, and 20% organic components based on the SeaSweep sea salt particle composition). The limits on the $M_{ET,j}$ summation are the size cuts of the IC samples (180 nm to 550 nm) to which the HR-AMS CEss is calibrated. The small number of salt particles larger than the 550 nm mobility Diameter cutoff of ET-AMS (< 3 cm^-3^ for NAAMES1 and < 6 cm^-3^ for NAAMES2) was not included. $M_{HRSS}$ is the HR-AMS sea salt mass, ${\mathrm{and}X}_{sweep}$ is the sea salt organic mass fraction observed during SeaSweep (Supplementary Table S3). ${CE}_{SS}$ = $M_{HRSS}$/$M_{ICSS}$ where$M_{ICSS}$ is the mass of IC sea salt, which is calculated from 3.26* Na^+^ to account for the mass of sodium chloride, magnesium sulfate and other inorganic salts present in seawater.

The activation diameter for the Estimated Salt at 0.1% supersaturation is 130 nm (Supplementary Table S6).

*HR-AMS Vaporizer Temperature Effects*

WACS2 sampled with SeaSweep deployed at five stations with a range of chlorophyll-a concentrations. Phytoplankton pigments (chlorophyll *a*, Chl-a) in seawater samples were collected from the R/V *Knorr* underway line (depth = 5m) and filtered through 25 mm glass-fiber filters (GF/F) with a nominal pore size of 700 nm under low vacuum pressure (< 5 psi) and dim light. Filters were placed in 100% methanol to extract at -20°C for 24 hr prior to measuring and the Chl-a was measured using a Turner 10AU fluorometer calibrated using pure Chl-a standard^18^.

Two HR-AMS instruments were operated simultaneously at different temperatures during WACS2 SeaSweep deployments. This approach increased the fraction of refractory sea salt particles and associated organic mass measured relative to a single instrument held at 650^o^C ^1,13,14,19^. The first AMS vaporizer was continuously held at approximately 560^o^C (referred to as AMS560), while the SP-AMS was set at approximately 660^o^C (referred to as AMShot) then increased to 700^o^C for 5 hours at station 3, and 800^o^C for 3 hours at station 5. CE_ss_ and high O/C organic mass fraction increase with the AMS vaporizer temperature (Figure S9). SeaSweep particles were also collected for FTIR analysis, but the samples were dehydrated to remove interference of sea salt hydrate bound water with the organic signal in the FTIR spectra^20^. The CE_org_ of the SeaSweep sea spray particles calculated from the FTIR organic mass concentration is 0.23 for the AMS560 and 0.18 for AMShot at 660^o^C. At higher vaporizer temperatures, the AMS measured a larger fraction of the IC-measured sea salt mass. The organic oxygenated mass fraction and O/C increased with vaporizer temperature. This supports the hypothesis that the lower volatility of salt particles accounts for the lower O/C measured by AMS (relative to the FTIR)^1^. However, the change in organic composition may also be due to differences in organic fragmentation at higher vaporizer temperatures or difference in organic composition in large particles (>700 nm) that are not measured by the AMS. SeaSweep sea spray particle detection varied at the different WACS2 stations with an apparent dependence on the measured Chl-a concentration, even though organic properties showed no dependence on Chl-a (Figure S9). These limitations of measuring sea salt particles by HR-AMS are addressed by calibration to filter-based IC measurements of sea salt.

Fourier Transform Infrared (FTIR) spectroscopy and AMS showed submicron particles contain two types of functional group composition, one with high and the other with low ratios of oxygen to carbon (O/C)^1,21^. Frossard et al.^1^ explicitly compared the organic chemical composition of SeaSweep marine particles using multiple measurement methods, including FTIR and AMS, and showed that the apparent discrepancy was due to the large fraction of refractory salt particles in the generated sea spray. Also, the AMS high O/C organic mass fraction is 11% lower than in the FTIR for SeaSweep sea spray particles, suggesting that the high O/C organic mass is more likely to be on refractory sea salt particles but the low O/C organic mass components are on particles with less salt. FTIR shows a greater fraction of high O/C organic components than the AMS for all stations, consistent with Frossard et al.^1^. Supplementary Figure S10c shows an increase in AMS high O/C OM fraction at higher vaporizer temperatures. The AMS high O/C OM fraction at higher vaporizer temperatures is still significantly lower than it is for the FTIR, as is expected given that even at higher vaporizer temperatures the CE of refractory sea salt is well below 0.1. Supplementary Figure S10b shows that the ratio of OM to sea salt decreases with greater vaporizer temperatures, with the exception of station 2, because the sea salt concentration increases more than the OM as vaporizer temperature was increased since sea salt was disproportionally on particles that were more refractory. Supplementary Figure S10a shows the ratio of OM is fairly consistent at stations 2 and 3 even when the vaporizer temperature is increased at station 3. Stations 4 and 5 have higher OM ratios when AMShot is at 660ºC, which could result from the higher particle mass concentration at these stations. At station 5, the OM ratio increases when the AMShot vaporizer temperature is increased from 660ºC to 800ºC, possibly due to the increase in vaporization of OM on sea salt particles.

*Hygroscopicity and CCN Calculations*

The hygroscopicity parameter is calculated from

$\kappa=\sum_{j} v_{j}\kappa_{j}$ (2)

where *v_j_* is the volume fraction of each component (j) and κ_j_ is the hygroscopicity parameter for the component. The four components used are in Supplementary Table S5. The volume fraction is calculated by multiplying the mass fraction (Supplementary Table S3) by the density (Supplementary Table S5) of each component.

The organic hygroscopicity in New Sulfate, Added Sulfate, MO, and LO particles is assumed to be 0.1, consistent with the range identified by Mochida et al.^22^ (Supplementary Table S6). The organic hygroscopicity for the Estimated Salt type was assumed to be 0.2 based on the high fraction of oxygenated mass fragments associated with sea spray particles^1^ (Supplementary Note). The organic hygroscopicity of the FF type was assumed to be 0.01, consistent with low O/C organic particles in urban areas^23^.

The resulting κ values (Supplementary Table S6) were used to calculate the minimum activation diameter (D_act_) for each particle type^24^, and the number of particles of each type that were larger than D_act_ were summed to give CCN at 0.1% supersaturation (Supplementary Figure S7). The size bin that included D_act_ was linearly interpolated so that only the fraction of particles in each bin with sizes greater than D_act_ were included. D_act_ for some of the particle types is below the ET-AMS lower cut off diameter (Supplementary Table S4). The particle range below the 180 nm ET-AMS cut off diameter are included but account for a small fraction of the CCN (Supplementary Figure S7).

*Inversion Strength Calculations*

The inversion strength was estimated by integrating the convective inhibition (CIN) over the inversion layer for $\mathrm{CIN}=\int_{z_{b}}^{z_{t}} -g*\frac{T_{v,ap}-T_{v}}{T_{v}}dz$ , where *z_t_* and *z_b_* are the top and bottom of the inversion layer, respectively, g is the acceleration due to gravity, *T_v_* is the virtual temperature in units of degrees Kelvin, calculated from the radiosonde measured temperature and relative humidity, and T_v,ap_ is the virtual temperature of a theoretical parcel that rises adiabatically. This definition of CIN is slightly different from the traditional definition where *z_t_* would instead be equivalent to the level of free convection. The bottom of the inversion layer is defined by a minimum in the temperature profile, just below a temperature increase in the inversion. Radiosonde profiles that showed evaporative cooling at cloud top are not included in the analysis because the minimum temperature does not accurately define the bottom of the inversion layer which makes CIN ill-bounded. The top of the inversion layer is defined by a maximum in the measured temperature just below the free troposphere, at which temperature decreases consistently with altitude. In cases with weak inversions, the vertical temperature profile does not have a minimum or maximum temperature at the inversion, but instead a change in slope at the top and bottom of the inversion. The second variable used to identify the inversion strength is the buoyancy jump, given by $\Delta b=g\frac{\Delta T_{v}}{T_{v}}$, where Δ*T_v_* is the change in the virtual potential temperature across the inversion layer.

Correlations of particle types with CIN are shown in Figure 4 and S11. The correlation of particle types with CIN are stronger than correlations with Δb (Figure 4, S11 and S12), consistent with the expectation that CIN is a more accurate representation of the inversion strength because it integrates across the inversion the difference between the temperature of an adiabatic parcel and the observed temperature, whereas Δb only depends on the temperature difference above and below the inversion. The CIN from the two radiosondes collected on this day are low (6 J kg^-1^ and 13 J kg^-1^), indicating a higher rate of entrainment from the free troposphere to the boundary layer.

Supplementary Table S1. Selected references on DMS as a marine aerosol source.

| Relevant Findings | Ref. | Location | Observations or Model |
| --- | --- | --- | --- |
| **Relationship between DMS and CCN** |  |  |  |
| DMS-derived sulfate aerosol account for most of the CCN in the remote marine boundary layer. | ^25^ | Global | Model plus Observations |
| MSA and CCN vary seasonally and have a non-linear relationship. | ^26^ | Cape Grim | Observations |
| DMS and CCN in boundary layer are strongly (non-linearly) correlated. | ^27^ | NE Pacific | Observations |
| CCN and DMS are correlated but relationship can be nonlinear because of SO_2_ sinks. | ^28^ | N/A | Model |
| CN correlates strongly with atmospheric DMS and DMS flux but weakly with CCN. | ^29^ | S. Atlantic | Observations |
| Modeled CN and CCN correlate with DMS flux; free tropospheric entrainment affects CN and CCN concentration in the marine boundary layer. | ^30^ | N/A | Model |
| **New Particle Formation from DMS Products** |  |  |  |
| The number of particles formed by homogeneous nucleation depends on the preexisting aerosol concentration. | ^31^ | N/A | Model |
| Particle number concentration increases rapidly after a decrease in particle surface area and increase in SO_2_ concentration. | ^32^ | NE Pacific | Observations |
| After precipitation, marine boundary layer aerosol particles can be replenished from new particles formed by nucleation if DMS concentrations are high. | ^33^ | N/A | Model |
| **Evidence of New Particle Formation in the Free Troposphere** |  |  |  |
| Vertical profiles of Aitken mode aerosol concentrations showed maximum values just above cloud tops. | ^34^ | NW and NE Pacific | Observations |
| Aerosol nucleation is observed above cloud top and downwind of cloud outflows. | ^35^ | N. Pacific | Observations |
| CN and CCN were replenished on time scales of 2-4 days with transported nuclei from the free troposphere after precipitation scavenging. | ^36^ | Christmas Island | Observations |
| Variability in marine boundary layer aerosol concentration is closely linked to changes in vertical transport. | ^37^ | NE Atlantic | Observations |
| Nucleation is observed in the free troposphere but not the marine boundary layer, and it is observed more frequently for particle surface area less than 5-10 µm^2^ cm^-3^. | ^38^ | Southern Ocean | Observations |
| CN concentration in the marine boundary layer is controlled by the rate of entrainment from the free troposphere in most conditions. | ^39^ | N/A | Model |
| Observed growth rates of new particles in the free troposphere cannot be explained by SO_2_ products and water vapor so other components must contribute to condensation. | ^40^ | NE Atlantic | Model/Observations |
| New sulfate particles do not form in the marine boundary layer but instead in the free troposphere and then are entrained downward. | ^41^ | Global | Model |
| Entrainment of nucleated sulfate particles from the free troposphere account for 43-65% of CCN, but only 7-20% in the winter; long range transport of marine CCN results in a time lag between CCN and DMS concentrations. | ^42^ | Southern Ocean | Model |
| 45% of marine boundary layer CCN (at 0.2%) are from nucleation that occurred in the free troposphere. | ^43^ | Global | Model |
| Sulfate particles from DMS mixed up to the free troposphere are a source of marine boundary layer CCN. | ^44^ | Tropical Pacific | Observations |

|  | **Correlations (r)** | | | | | | |  |
| --- | --- | --- | --- | --- | --- | --- | --- | --- |
| **Particle Types** | **Radon** | **Black Carbon** | **fC_4_H_9_** | **fC_2_H_4_O_2_** | **MSA^1^** | **CIN** | **DMS: Atmospheric**  **(Seawater)** | **References for Comparison** |
| **NAAMES1** |  |  |  |  |  |  |  |  |
| Non-Marine |  |  |  |  |  |  |  |  |
| MO | **0.30** | 0.05 | **0.32** | **0.32** | - | **0.40** | 0.05 (-0.27) | ^45^ (OOA-1), ^46^ (LV-OOA) |
| MC | 0.05 | 0.12 | 0.04 | 0.04 | - | **0.48** | 0.22 (-0.16) | ^47^ (cluster82), ^48^ (IEPOX OA) |
| LO | **0.40** | -0.06 | **0.40** | **0.40** | - | **0.81** | 0.15 (-0.22) | ^45^ (OOA-2), ^46^ (SV-OOA) |
| FF | 0.07 | 0.23 | 0.11 | 0.11 | - | **0.59** | 0.01 (-0.11) | ^3^ (HOA), ^2^ (HOA) |
| Added Sulfate | 0.09 | -0.01 | 0.16 | 0.16 | - | **-0.42** | 0.00 (-0.13) | ^49^ (MSA-OA) |
| New Sulfate | **-0.44** | -0.05 | **-0.46** | **-0.45** | - | **-0.74** | -0.17 (**0.37**) | This study |
| **NAAMES2** |  |  |  |  |  |  |  |  |
| Non-Marine |  |  |  |  |  |  |  |  |
| MO | -0.20 | 0.04 | 0.13 | **0.49** | **-0.60** | **0.62** | **-0.51** (-0.28) | ^45^ (OOA-1), ^46^ (LV-OOA) |
| MC | **0.55** | -0.06 | 0.04 | 0.08 | 0.06 | **0.61** | 0.03 (0.29) | ^47^ (cluster82), ^48^ (IEPOX OA) |
| LO | **0.49** | 0.01 | 0.16 | **0.43** | **-0.63** | **0.83** | **-0.56** (-0.29) | ^45^ (OOA-2), ^46^ (SV-OOA) |
| FF | 0.01 | **0.48** | **0.70** | 0.04 | **-0.36** | **0.52** | -0.03 (0.07) | ^3^ (HOA), ^2^ (HOA) |
| Added Sulfate | **-0.25** | -0.24 | 0.15 | **-0.48** | **0.60** | -0.16 | **0.50** (**0.32**) | ^49^ (MSA-OA) |
| New Sulfate | **-0.32** | -0.15 | 0.16 | **-0.28** | **0.30** | **-0.81** | **0.28** (0.01) | This study |
|  |  |  |  |  |  |  |  |  |

Supplementary Table S2. NAAMES1 and NAAMES2 single particle aerosol types identified by ET-AMS and their compositions and correlations to tracers. The characteristic m/z peaks for each particle type (shown in Supplemental Figure S1) are listed in order of abundance. WACS2 is not included because there were not enough measurements to correlate time series. Weak correlations (|r| >= 0.25 and |r| < 0.50), moderate correlations (|r| >= 0.50 and |r| < 0.80) and strong correlations (|r| >= 0.80) are in bold. Correlations are for all ambient measurements, except for MSA and DMS, which were only correlated with measurements in clean marine air masses.

^1^ MSA measurements in NAAMES1 were below detection limit so no correlations are reported.

Supplementary Table S3. The high m/z peaks and measured mass fractions of organic, sulfate, nitrate and sea salt components in the centroids of the clusters of single-particle mass spectra.

| **Particle Types** | **Organic (%)** | **Sulfate^1^ (%)** | **Nitrate (%)** | **Salt (%)** | **Major m/z Fragments** |
| --- | --- | --- | --- | --- | --- |
| **WACS2** |  |  |  |  |  |
| Non-Marine |  |  |  |  |  |
| More Oxidized organics (MO) | 80.7 | 7.6 | 8.6 | 3.0 | 44, 29, 43 |
| Continental Mixed (MC) | 79.0 | 15.1 | 4.6 | 1.4 | 44, 29, 48 |
| Less Oxidized organics (LO) | 79.5 | 10.7 | 4.8 | 5.0 | 43, 29, 41 |
| Fossil Fuel combustion (FF) | - | - | - | - | - |
| Added Sulfate | 57.8 | 35.3 | 4.2 | 2.7 | 48, 64, 29 |
| New Sulfate | 37.9 | 60.6 | 1.0 | 0.5 | 48, 64, 80 |
| Estimated Salt | - | - | - | - | - |
| **NAAMES1** |  |  |  |  |  |
| Non-Marine |  |  |  |  |  |
| More Oxidized organics (MO) | 85.5 | 9.6 | 4.4 | 0.5 | 44, 43, 29 |
| Continental Mixed (MC) | 65.5 | 31.2 | 2.7 | 0.6 | 44, 64, 48 |
| Less Oxidized organics (LO) | 74.5 | 20.9 | 3.7 | 0.9 | 43, 44, 55 |
| Fossil Fuel combustion (FF) | 93.1 | 5.7 | 0.7 | 0.5 | 43, 57, 55 |
| Added Sulfate | 60.4 | 34.8 | 4.1 | 0.7 | 48, 64, 43 |
| New Sulfate | 35.3 | 62.3 | 1.9 | 0.5 | 64, 48, 80 |
| Estimated Salt | 20.8 | 6.0 | 1.5 | 71.7 | 35, 36, 23 |
| **NAAMES2** |  |  |  |  |  |
| Non-Marine |  |  |  |  |  |
| More Oxidized organics (MO) | 81.1 | 15.1 | 3.4 | 0.5 | 44, 43, 29 |
| Continental Mixed (MC) | 71.3 | 22.5 | 5.9 | 0.3 | 44, 64, 29 |
| Less Oxidized organics (LO) | 81.8 | 12.0 | 5.7 | 0.5 | 43, 44, 29 |
| Fossil Fuel combustion (FF) | 92.8 | 5.7 | 0.8 | 0.6 | 57, 43, 55 |
| Added Sulfate | 55.2 | 42.2 | 2.1 | 0.5 | 64, 48, 44 |
| New Sulfate | 35.7 | 62.7 | 1.3 | 0.3 | 64, 48, 80 |
| Estimated Salt^2^ | 20.8 | 6.0 | 1.5 | 71.7 | 35, 36, 23 |

^1^Sulfate mass fraction is corrected as described in Supplemental Note.

^2^ The Estimated Salt particle type for NAAMES1 is used for NAAMES2 because the ROIs for the ET-AMS were not optimized to collect sea salt particles during NAAMES2.

Supplementary Table S4. Cumulative number of single particle measurements for WACS2, NAAMES1 and NAAMES2.

| **Statistic** | **WACS2 (LS-AMS)** | **NAAMES1 (ET-AMS)** | **NAAMES2 (ET-AMS)** |
| --- | --- | --- | --- |
| Total triggers | 1471 | 2460399 | 2392300 |
| Prompt particles^1^ | 362 | 85909 | 159359 |
| D_min_ (nm)^2^ | 400 | 145 | 180 |

^1^Prompt particles are those that have above the minimum S/N of 5 and greater than 10 ions measured within 200 µs of impacting the vaporizer surface.

^2^The minimum mobility particle diameter is defined as the smallest diameter with a 5% collection efficiency.

Supplementary Table S5. Chemical composition and physical properties used for calculating hygroscopicity from AMS measured components.

| **AMS Ions** | **Molecular Composition** | **Density (g cm^-3^)** | **Hygroscopicity (κ)** |
| --- | --- | --- | --- |
| Organic | - | 1 | (0.01-0.2, see Supplementary Table S6) |
| Sulfate | NH_4_HSO_4_ | 1.77 | 0.84 |
| Nitrate | NH_4_NO_3_ | 1.72 | 0.78 |
| Salt | NaCl | 2.16 | 1.33 |

Supplementary Table S6. The ambient hygroscopicity, activation diameter and fraction of particles that contain salt ions for each aerosol type, based on the chemical composition in Supplementary Table S3. The non-marine particle types, More Oxygenated (MO), Less Oxygenated (LO), Mixed Continental (MC), and Fossil Fuel (FF), are discussed in the Supplementary Note.

| **Particle Types** | **Organic Hygroscopicity^2^ (κ_org_)** | **Total Hygroscopicity (κ)** | **Minimum Activation Diameter^3^ (nm)** | **Salt Containing**  **(m/z 35+ m/z 36) Fraction** |
| --- | --- | --- | --- | --- |
| **NAAMES1** |  |  |  |  |
| Non-Marine |  |  |  |  |
| MO | 0.2 | 0.27 | 198 | 0.23 |
| MC | 0.1 | 0.31 | 189 | 0.12 |
| LO | 0.1 | 0.25 | 203 | 0.14 |
| FF | 0.01 | 0.05 | 346 | 0.13 |
| Added Sulfate | 0.1 | 0.34 | 183 | 0.12 |
| New Sulfate | 0.1 | 0.56 | 155 | 0.12 |
| Estimated Salt^1^ | 0.2 | 0.95 | 130 | 1.00 |
| **NAAMES2** |  |  |  |  |
| Non-Marine |  |  |  |  |
| MO | 0.2 | 0.29 | 193 | 0.22 |
| MC | 0.1 | 0.26 | 200 | 0.19 |
| LO | 0.1 | 0.20 | 219 | 0.19 |
| FF | 0.01 | 0.05 | 346 | 0.16 |
| Added Sulfate | 0.1 | 0.38 | 177 | 0.18 |
| New Sulfate | 0.1 | 0.55 | 156 | 0.15 |
| Estimated Salt^4^ | 0.2 | 0.95 | 130 | 0.86 |

^1^ The sea salt particle type is based on ET-AMS single particle chemical composition of SeaSweep sea salt particles measured.

^2^ The organic hygroscopicity for FF is based on Sanchez et al.^50^, MO is based on Chang et al.^51^. The organic hygroscopicity of Estimated Salt is consistent with Frossard et al.^22^ and Quinn et al.^17^.

^3^ The minimum activation diameter is the mobility diameter and is calculated for a supersaturation of 0.1%.^24^

^4^ The Estimated Salt particle type for NAAMES1 is used for NAAMES2 because the ROIs for the ET-AMS were not optimized to collect sea salt particles during NAAMES2.


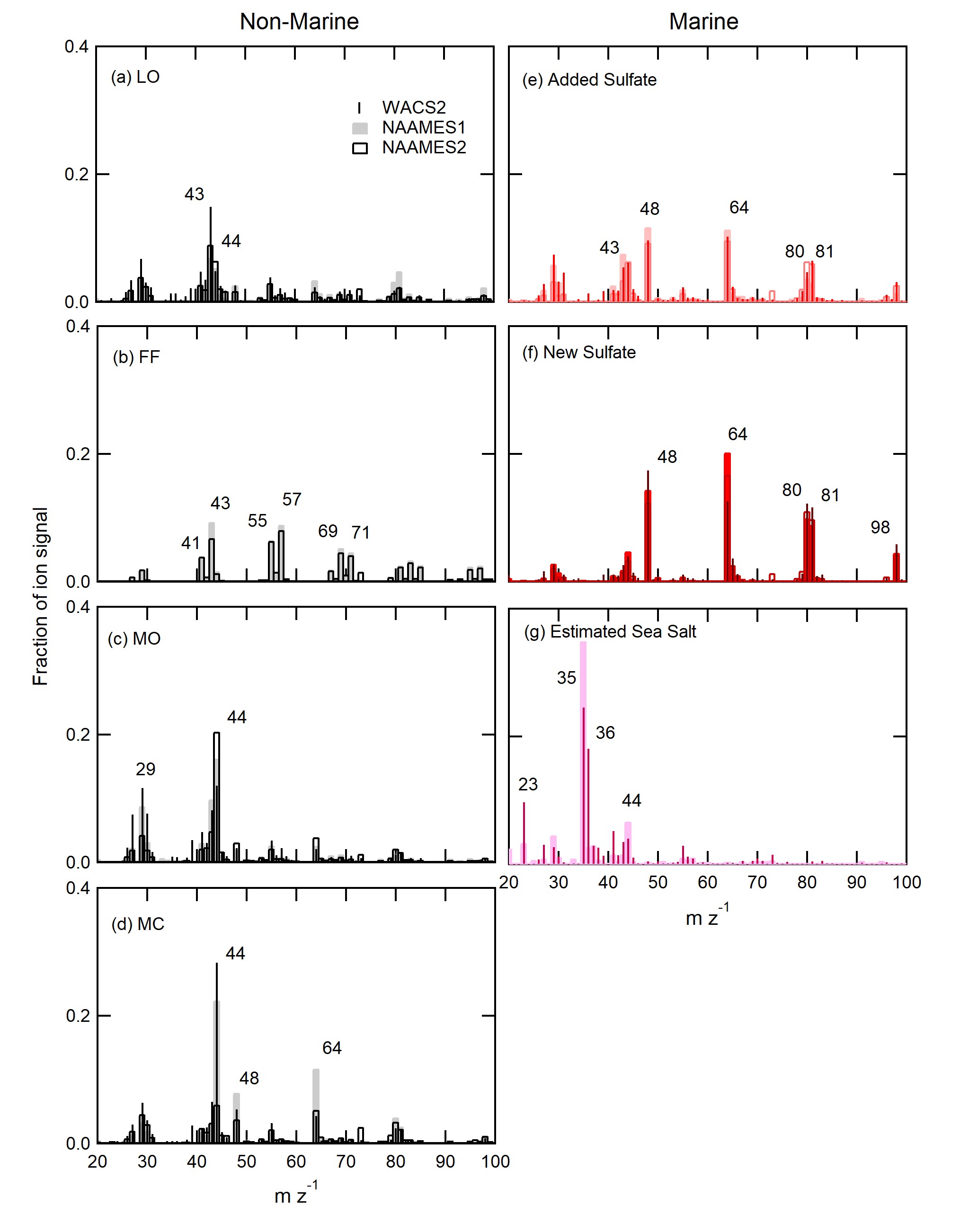


Supplementary Figure S1. The average mass spectra for particle types from LS-AMS during WACS2 and the ET-AMS during NAAMES1 and NAAMES2. All mass spectra are from ambient measurements except for the Estimated Salt type, which is from SeaSweep measurements. There are no mass spectra for the FF particle type in WACS2 because it was not measured in that project. There are no mass spectra for the Estimated Salt particle type in NAAMES2 because the ET-AMS regions of interest did not include m/z 23, 35 or 58.


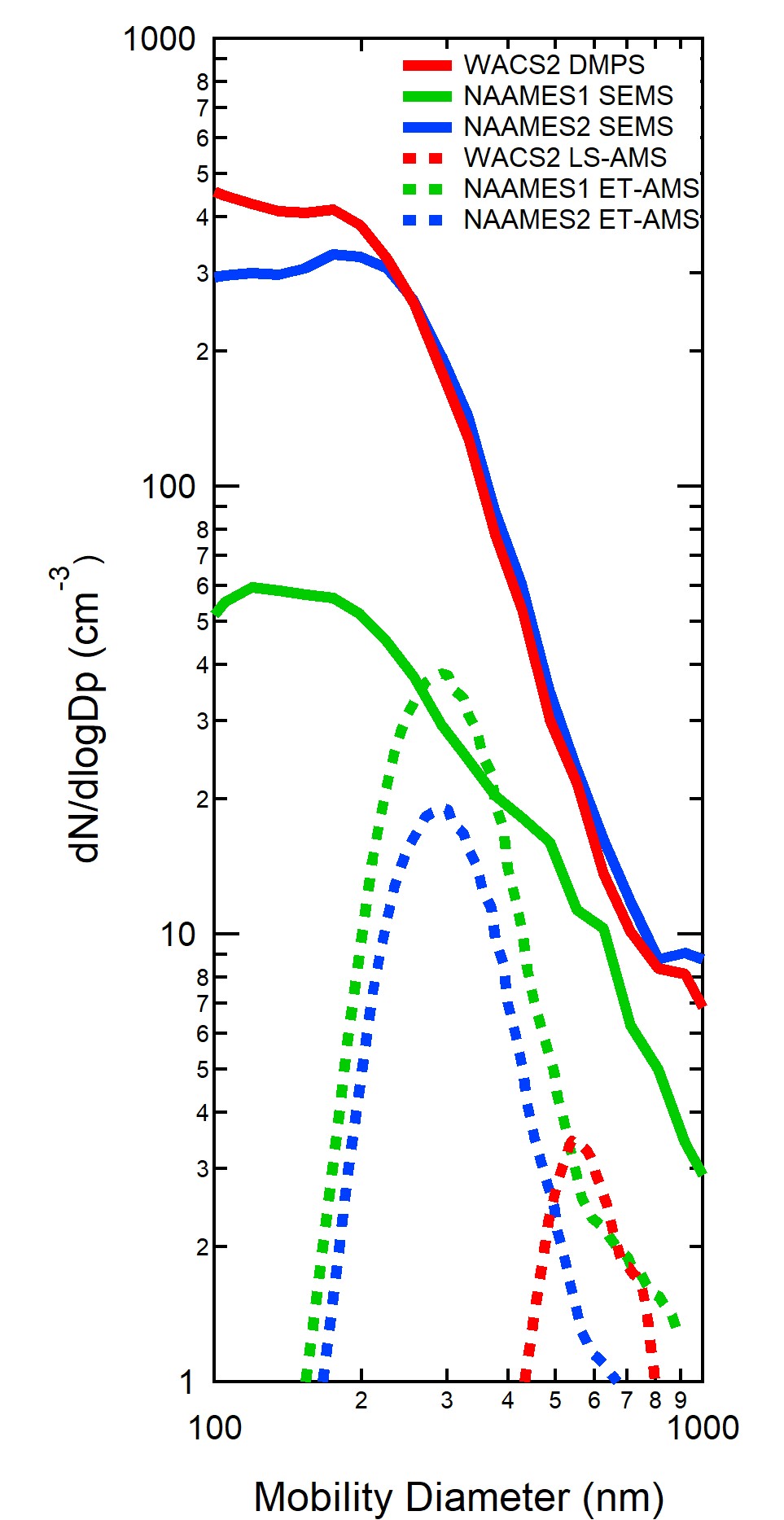


Supplementary Figure S2. Measurement range for LS-AMS and ET-AMS are compared to averaged SEMS and DMPS size distributions for clean marine conditions. The AMS aerodynamic diameter is converted to mobility diameter to compare with the SEMS (WACS2) or DMPS (NAAMES1 and NAAMES2)^52^. Campaign average particle densities derived from AMS particle time of flight, and SEMS or DMPS measurements are 1.26, 1.27, and 1.33 g cm^-3^ for WACS2, NAAMES1, and NAAMES2, respectively.


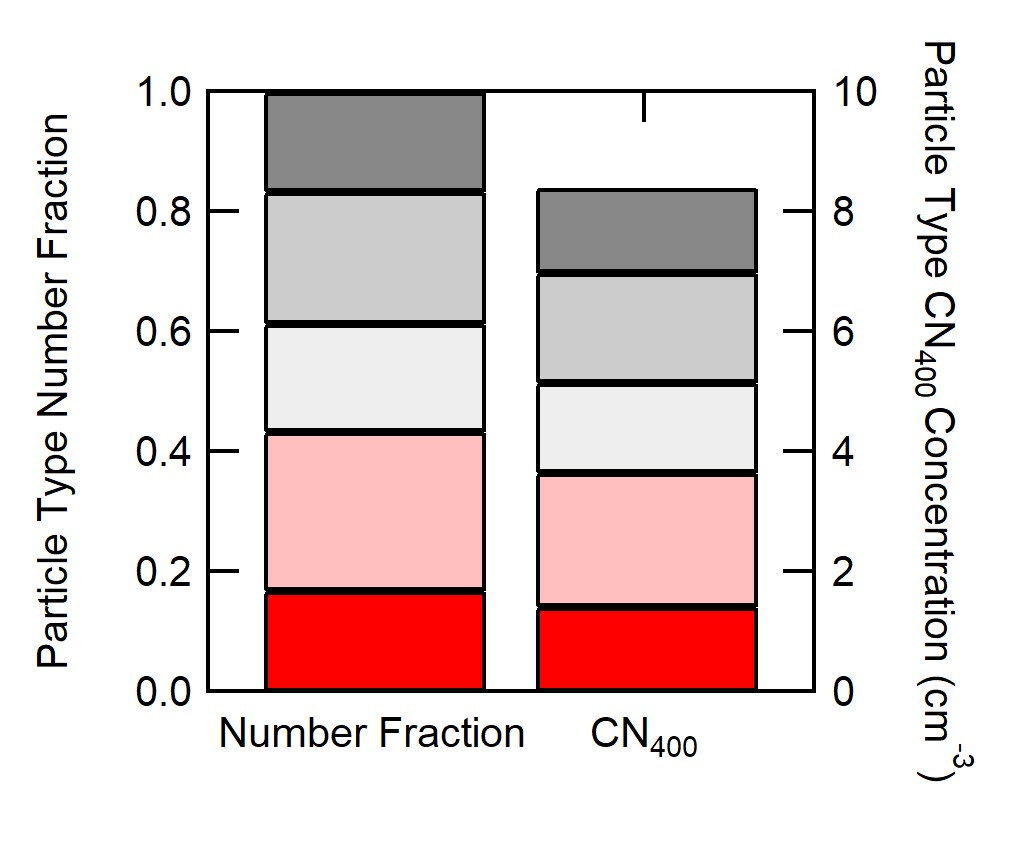


Supplementary Figure S3. WACS2 non-refractory LS-AMS particle type number fraction and CN greater than the LS-AMS minimum cut diameter of 400 nm (CN_400_) for clean marine periods. WACS2 included only limited time for sampling ambient air and so only four times were available for LS-AMS measurements (more than 3 hr).


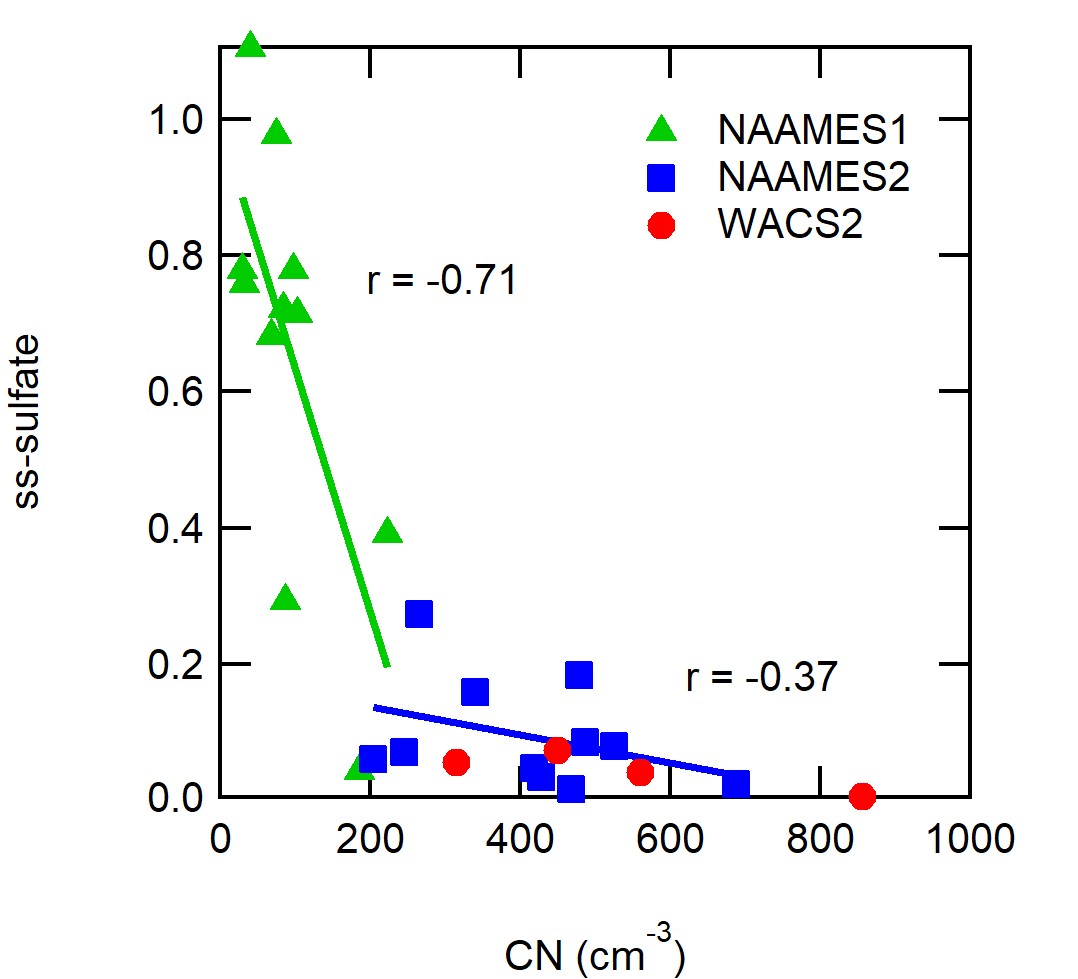


Supplementary Figure S4. The ss-sulfate fraction, calculated from measurements of IC sodium, chloride and sulfate, is compared to condensation nuclei (CN) concentrations for each campaign during clean marine periods. Linear regressions are shown for NAAMES1 and NAAMES2, which have correlation coefficients of -0.71 and -0.37, respectively.

Supplementary Figure S5. The ss-sulfate fraction is calculated from sub 1.1 µm measurements of IC sodium and sulfate. Total rain accumulation is calculated by integrating the rain accumulation from six hours before the IC filter sample start time to the filter sample end time for the NAAMES2 and WACS2 campaigns.


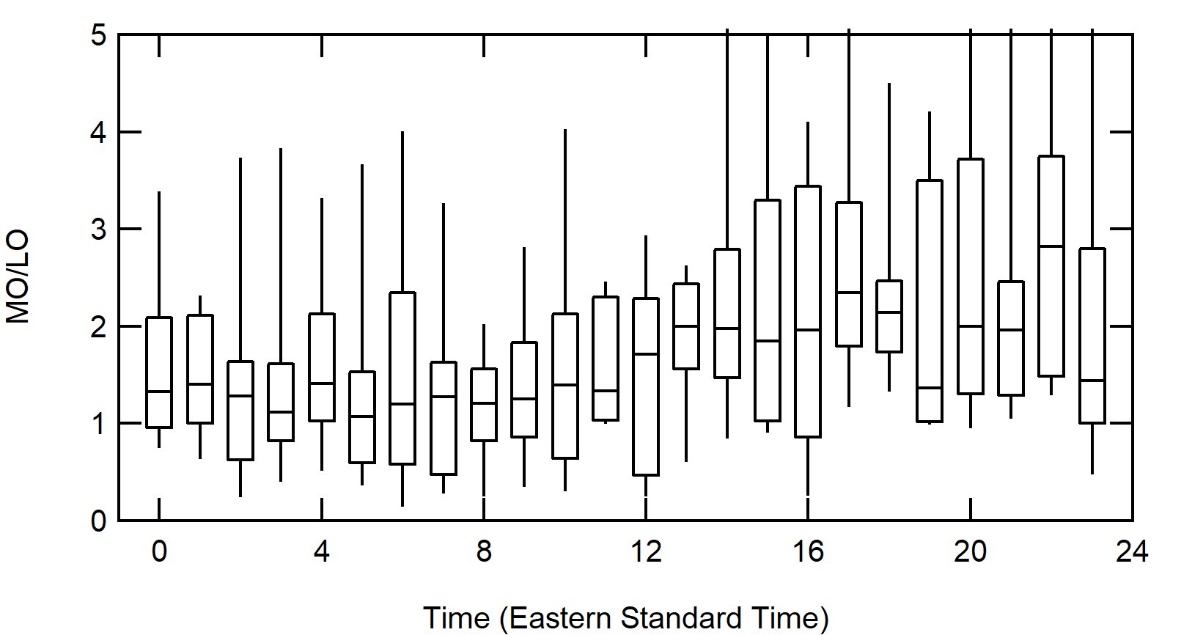


Supplementary Figure S6. Diurnal averaged number ratio of MO to LO particles for clean marine periods during NAAMES2. There are insufficient measurements of MO and LO during WACS2 and NAAMES1 so they are not shown.


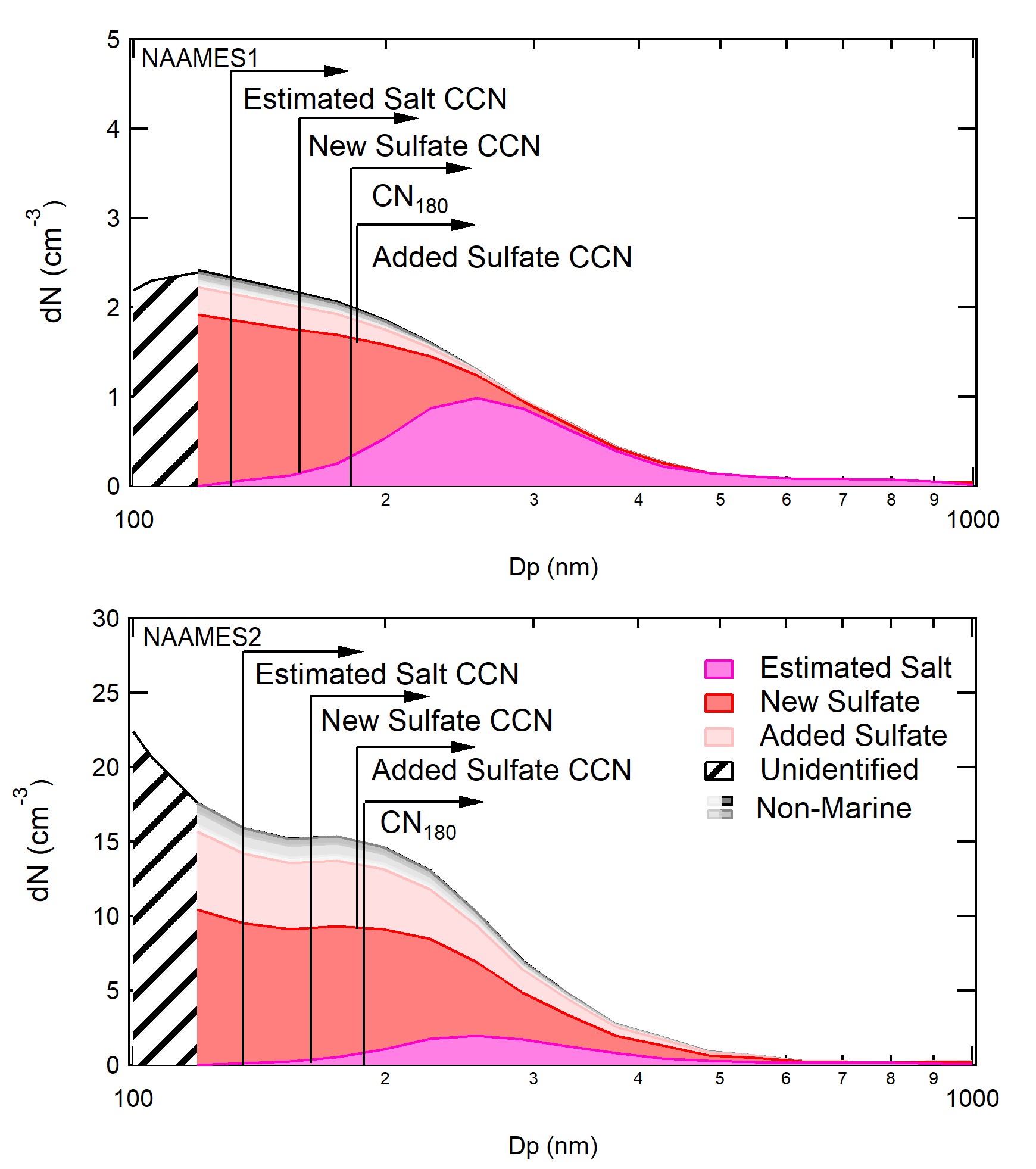


Supplementary Figure S7. Particle type size distributions for sample NAAMES1 and NAAMES2 cases. Black arrows identify the 0.1% supersaturation activation diameters for the Estimated Salt, New Sulfate, and Added Sulfate types.
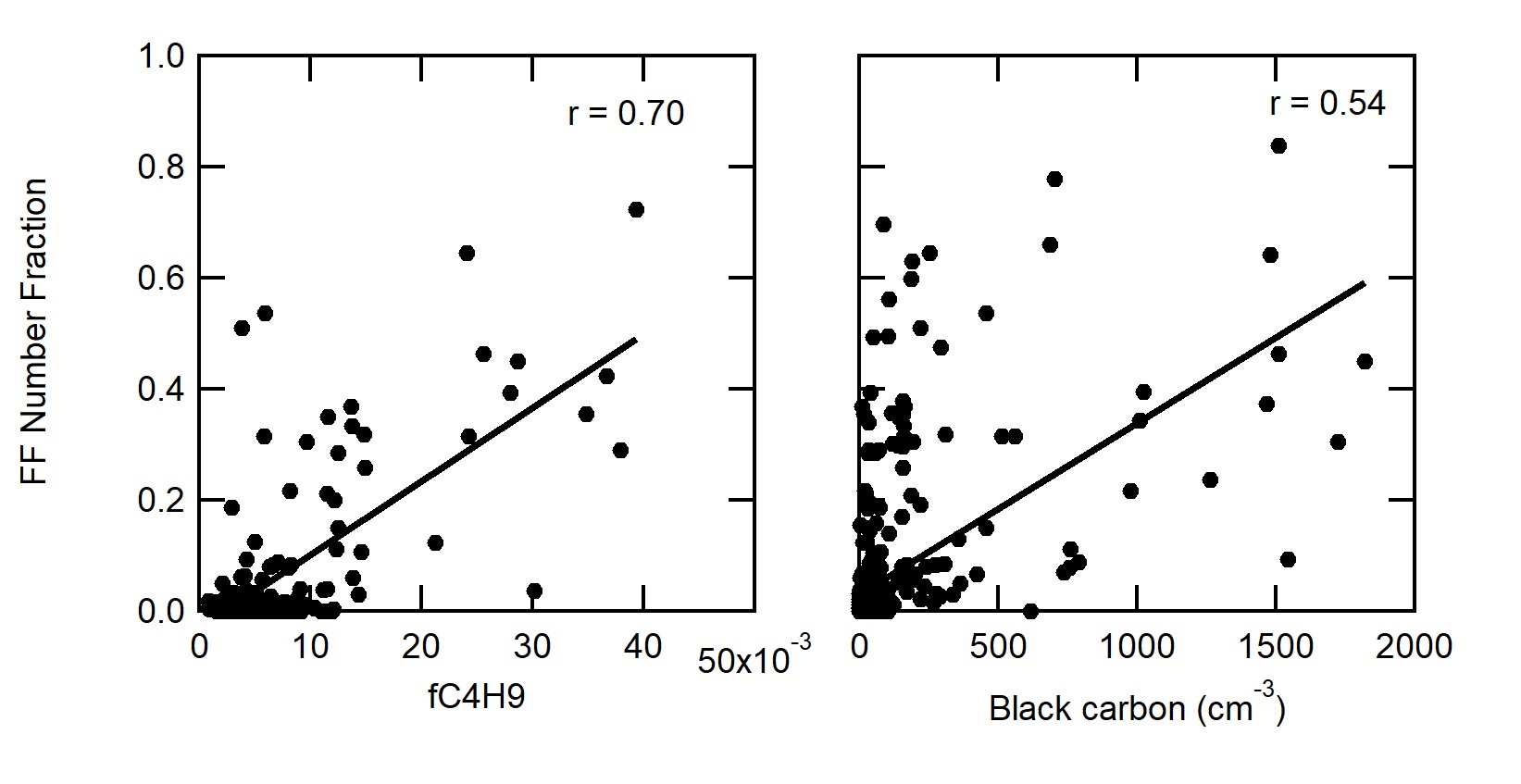


Supplementary Figure S8. The correlation of the fraction of the FF particle type to AMS organic fraction of C_4_H_9_ (a fossil fuel combustion tracer; left) and to black carbon number concentration (right) for NAAMES2. WACS2 LS-AMS measurements did not have FF particles and NAAMES1 contained too few FF particle measurements for correlation so are excluded.


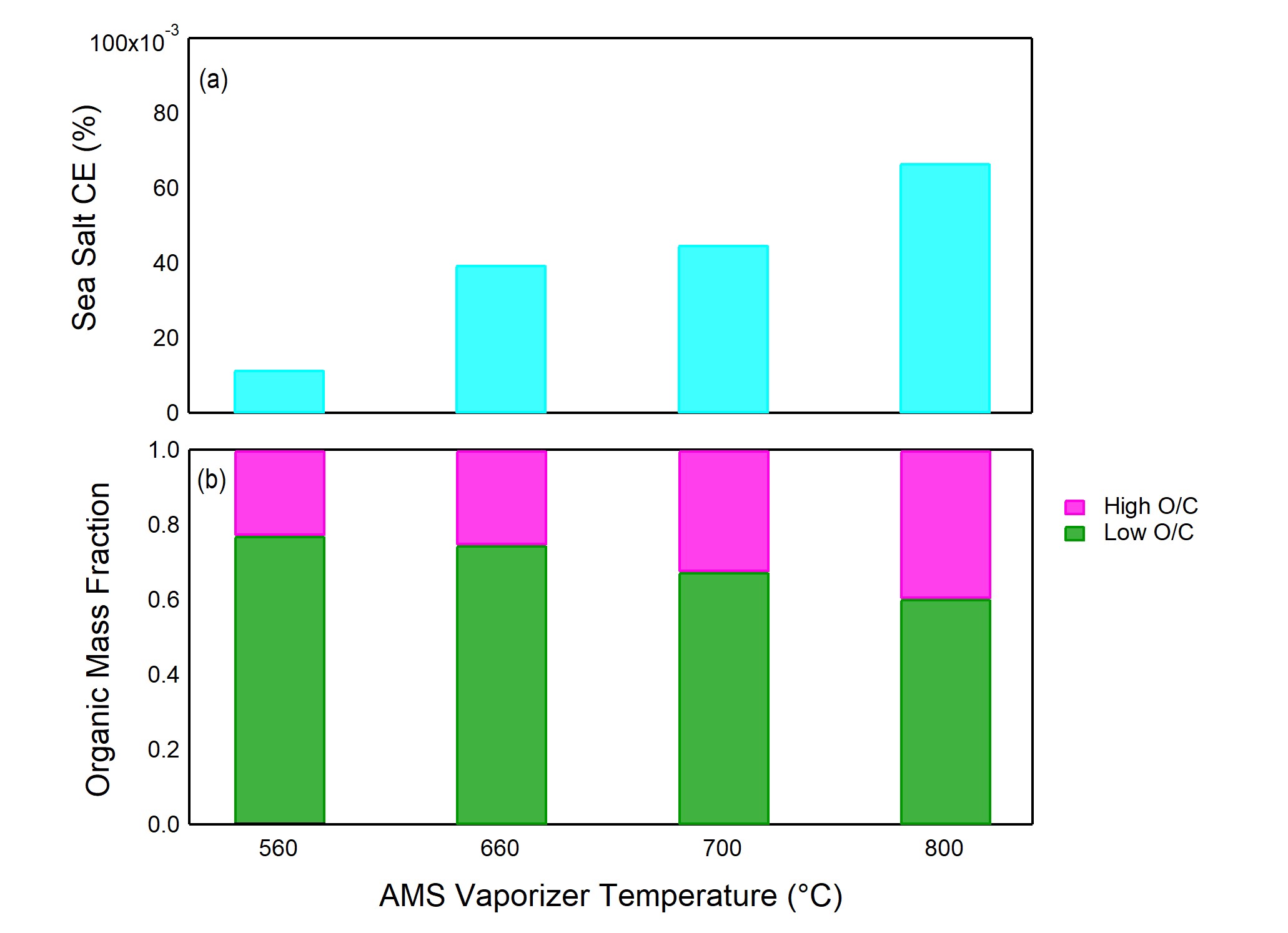
Supplementary Figure S9. Dependence of WACS2 HR-AMS mea­­­surements of SeaSweep particles on vaporizer temperature as shown by (a) the collection efficiency of sea salt calculated from IC measurements of sodium and chloride^1^ and (b) the organic mass fraction composition, where high O/C organic mass includes mass fragment groups C_X_H_Y_O and C_X_H_Y_O_>1_ and low O/C mass includes mass fragment group C_X_H_Y_.


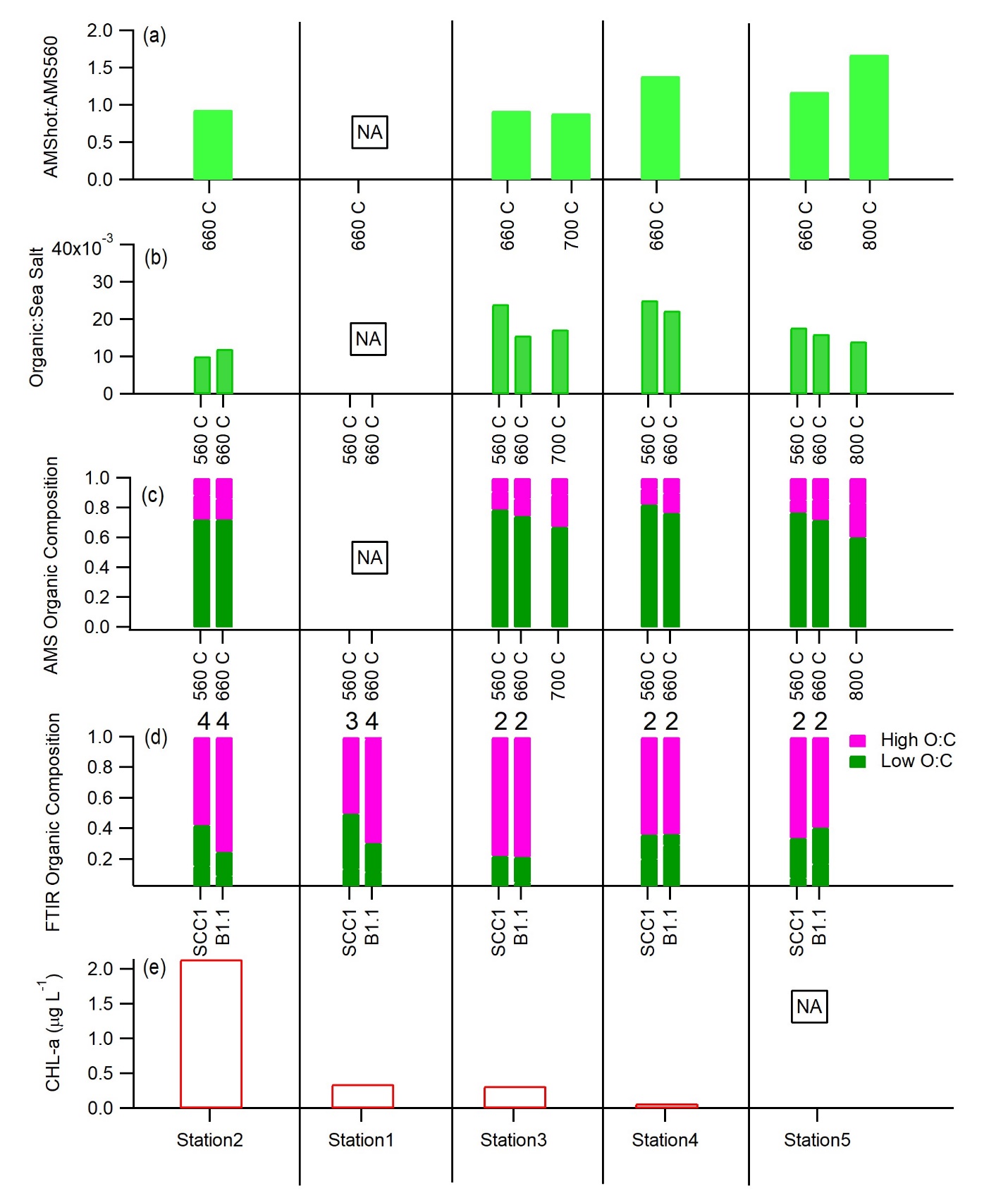


Supplementary Figure S10. For each SeaSweep station and each AMShot vaporizer temperature, (a) the ratio of the AMS organic mass, (b) the ratio of organic mass to sea salt mass, (c) the high and low O/C organic mass fraction, (d) FTIR high and low O/C organic mass fraction for particles collected after a 1 µm sharp cut cyclone (SCC1) or a 1.1 µm cut Berner impactor (B1.1), and (e) Chl-a concentration. The number of FTIR samples is given above each bar in (d).


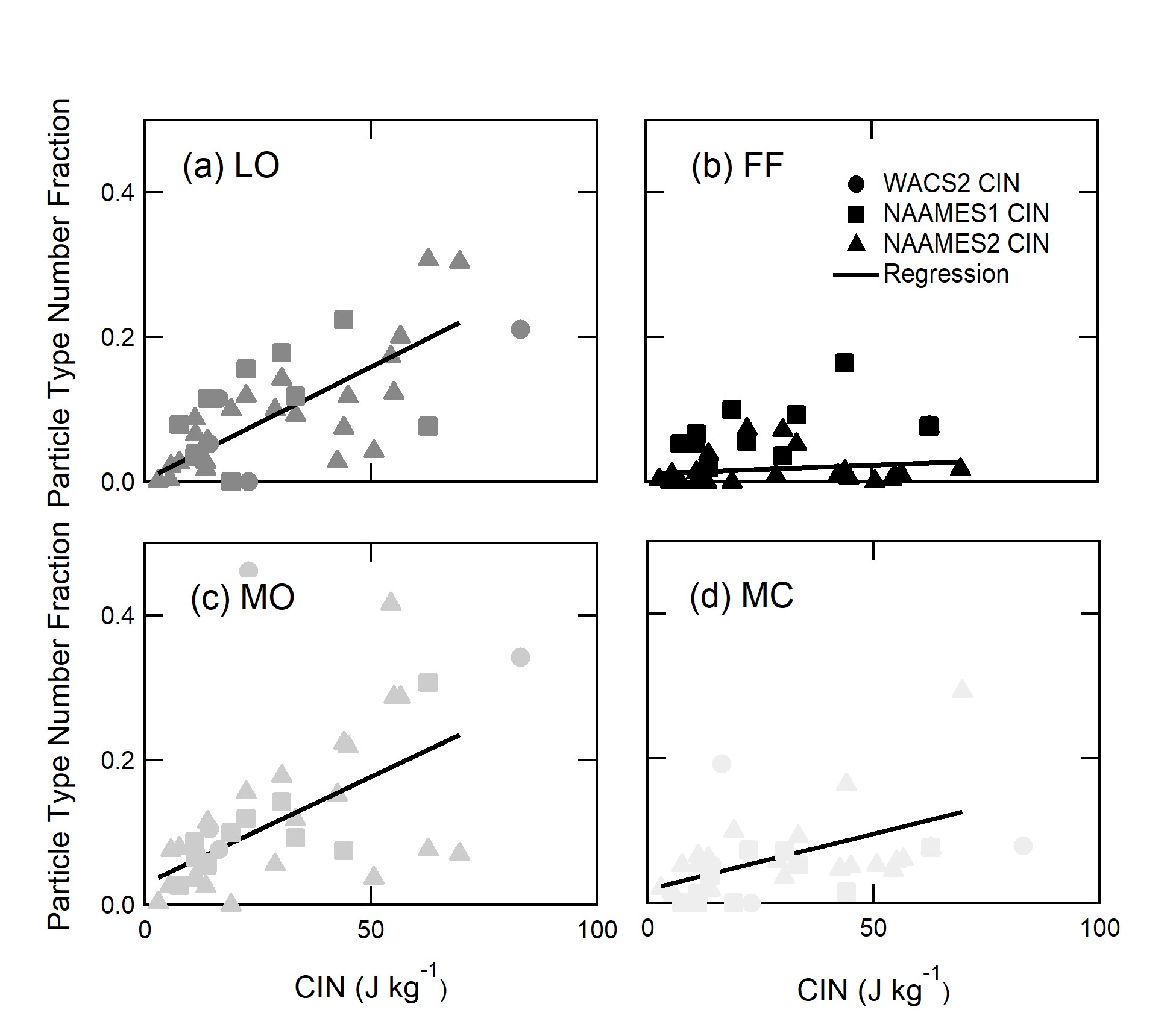


Figure S11. Dependence of WACS2 LS-AMS and NAAMES1 and NAAMES2 ET-AMS particle number fractions during clean marine conditions on CIN calculated from radiosonde measurements. Pearson correlation coefficients for NAAMES1 and NAAMES2 for CIN are (a) 0.77, (b) 0.21, (c) 0.58, and (d) 0.54 for the non-marine types. Added Sulfate and New Sulfate are included in Figure 4.


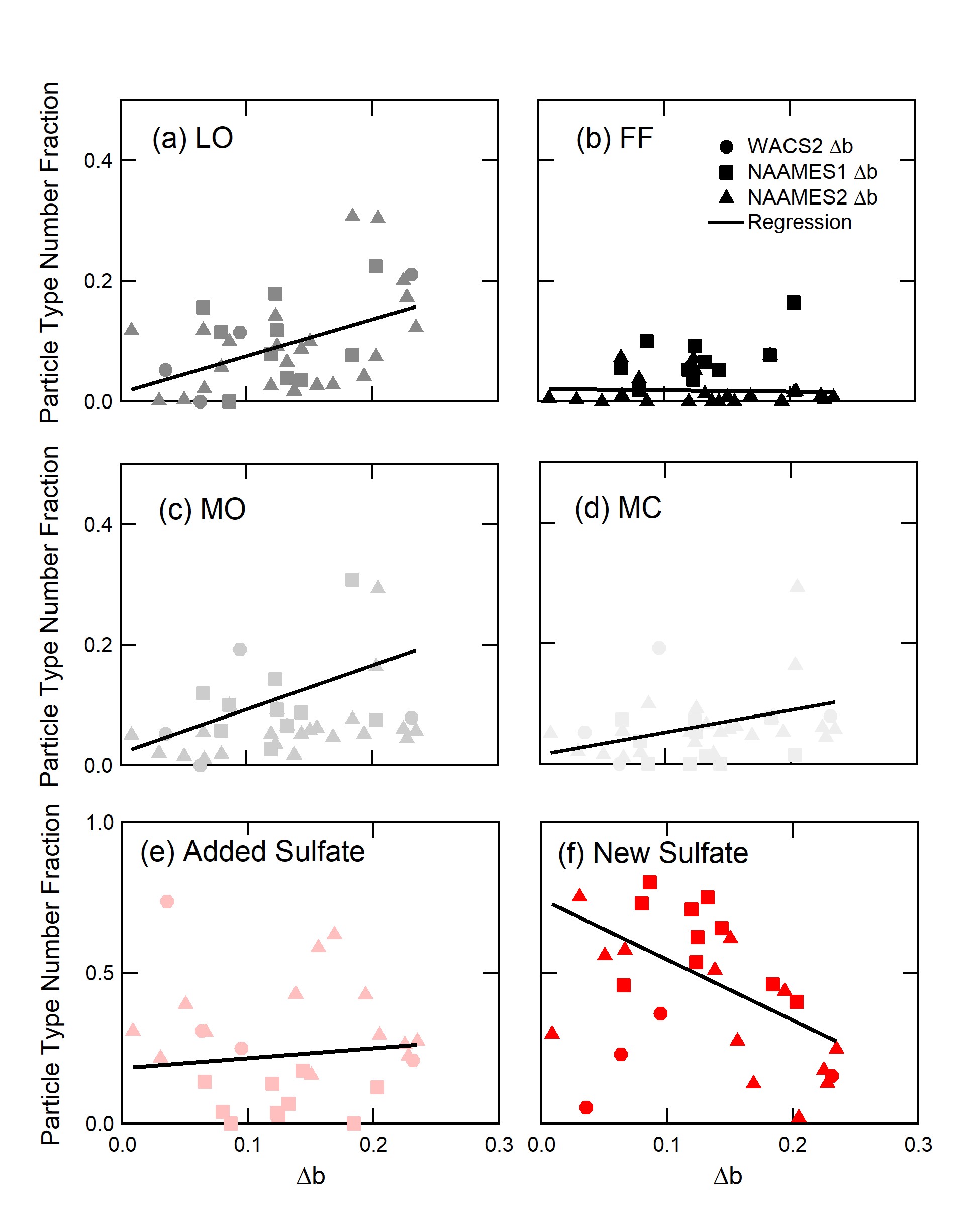


Figure S12. Dependence of WACS2 LS-AMS and NAAMES1 and NAAMES2 ET-AMS particle number fractions during clean marine conditions on Δb calculated from radiosonde measurements. Pearson correlation coefficients for NAAMES1 and NAAMES2 for Δb are (a) 0.47 for LO, (b) -0.06 for FF, (c) 0.44 for MO, (d) 0.41 for MC, (e) 0.01 for the Added Sulfate, and (f) -0.58 for the New Sulfate particles.

References

1 Frossard, A. A., Russell, L. M., Massoli, P., Bates, T. S. & Quinn, P. K. Side-by-Side Comparison of Four Techniques Explains the Apparent Differences in the Organic Composition of Generated and Ambient Marine Aerosol Particles. *Aerosol Science and Technology* **48**, V-X, doi:10.1080/02786826.2013.879979 (2014).

2 Zhang, Q. *et al.* Understanding atmospheric organic aerosols via factor analysis of aerosol mass spectrometry: a review. *Analytical and Bioanalytical Chemistry* **401**, 3045-3067, doi:10.1007/s00216-011-5355-y (2011).

3 Lanz, V. A. *et al.* Source apportionment of submicron organic aerosols at an urban site by factor analytical modelling of aerosol mass spectra. *Atmospheric Chemistry and Physics* **7**, 1503-1522 (2007).

4 Lee, A. K. Y., Willis, M. D., Healy, R. M., Onasch, T. B. & Abbatt, J. P. D. Mixing state of carbonaceous aerosol in an urban environment: single particle characterization using the soot particle aerosol mass spectrometer (SP-AMS). *Atmospheric Chemistry and Physics* **15**, 1823-1841, doi:10.5194/acp-15-1823-2015 (2015).

5 Liu, S., Russell, L. M., Sueper, D. T. & Onasch, T. B. Organic particle types by single-particle measurements using a time-of-flight aerosol mass spectrometer coupled with a light scattering module. *Atmospheric Measurement Techniques* **6**, 187-197, doi:10.5194/amt-6-187-2013 (2013).

6 Cross, E. S. *et al.* Single particle characterization using a light scattering module coupled to a time-of-flight aerosol mass spectrometer. *Atmospheric Chemistry and Physics* **9**, 7769-7793 (2009).

7 Dorr, H. & Munnich, K. O. SOIL RN-222 AS A TRACER FOR GAS-TRANSPORT IN THE UNSATURATED SOIL ZONE. *Chemical Geology* **70**, 97-97, doi:10.1016/0009-2541(88)90469-x (1988).

8 Bates, T. S. *et al.* Boundary layer aerosol chemistry during TexAQS/GoMACCS 2006: Insights into aerosol sources and transformation processes. *Journal of Geophysical Research-Atmospheres* **113**, doi:10.1029/2008jd010023 (2008).

9 Zhang, Q. *et al.* Deconvolution and quantification of hydrocarbon-like and oxygenated organic aerosols based on aerosol mass spectrometry. *Environmental Science & Technology* **39**, 4938-4952, doi:10.1021/es048568l (2005).

10 Price, D. J. *et al.* More unsaturated, cooking-type hydrocarbon-like organic aerosol particle emissions from renewable diesel compared to ultra low sulfur diesel in at-sea operations of a research vessel. *Aerosol Science and Technology* **51**, 135-146, doi:10.1080/02786826.2016.1238033 (2017).

11 Crippa, M. *et al.* Organic aerosol components derived from 25 AMS data sets across Europe using a consistent ME-2 based source apportionment approach. *Atmospheric Chemistry and Physics* **14**, 6159-6176, doi:10.5194/acp-14-6159-2014 (2014).

12 Shank, L. M. *et al.* Organic matter and non-refractory aerosol over the remote Southeast Pacific: oceanic and combustion sources. *Atmospheric Chemistry and Physics* **12**, 557-576, doi:10.5194/acp-12-557-2012 (2012).

13 Frossard, A. A. *et al.* Sources and composition of submicron organic mass in marine aerosol particles. *Journal of Geophysical Research-Atmospheres* **119**, 12977-13003, doi:10.1002/2014jd021913 (2014).

14 Bates, T. S. *et al.* Measurements of ocean derived aerosol off the coast of California. *Journal of Geophysical Research-Atmospheres* **117**, 13, doi:10.1029/2012jd017588 (2012).

15 Holland, H. D. *The Chemistry of the Atmosphere and Oceans*. (Jon Wiley 1978).

16 Quinn, P. K., Coffman, D. J., Kapustin, V. N., Bates, T. S. & Covert, D. S. Aerosol optical properties in the marine boundary layer during the First Aerosol Characterization Experiment (ACE 1) and the underlying chemical and physical aerosol properties. *Journal of Geophysical Research-Atmospheres* **103**, 16547-16563, doi:10.1029/97jd02345 (1998).

17 Quinn, P. K. *et al.* Contribution of sea surface carbon pool to organic matter enrichment in sea spray aerosol. *Nature Geoscience* **7**, 228-232, doi:10.1038/ngeo2092 (2014).

18 Coad, T. Chlorophyll a data collected during the SIPEX II voyage of the Aurora Australis, 2012. *Australian Antarctic Data Centre* (2014).

19 Keene, W. C. *et al.* Chemical and physical characteristics of nascent aerosols produced by bursting bubbles at a model air-sea interface. *Journal of Geophysical Research-Atmospheres* **112**, doi:10.1029/2007jd008464 (2007).

20 Frossard, A. A. & Russell, L. M. Removal of Sea Salt Hydrate Water from Seawater-Derived Samples by Dehydration. *Environmental Science & Technology* **46**, 13326-13333, doi:10.1021/es3032083 (2012).

21 Russell, L. M., Hawkins, L. N., Frossard, A. A., Quinn, P. K. & Bates, T. S. Carbohydrate-like composition of submicron atmospheric particles and their production from ocean bubble bursting. *Proceedings of the National Academy of Sciences of the United States of America* **107**, 6652-6657, doi:10.1073/pnas.0908905107 (2010).

22 Mochida, M. *et al.* Hygroscopicity and cloud condensation nucleus activity of marine aerosol particles over the western North Pacific. *Journal of Geophysical Research-Atmospheres* **116**, doi:10.1029/2010jd014759 (2011).

23 Petters, M. D., Kreidenweis, S. M. & Ziemann, P. J. Prediction of cloud condensation nuclei activity for organic compounds using functional group contribution methods. *Geoscientific Model Development* **9**, 111-124, doi:10.5194/gmd-9-111-2016 (2016).

24 Petters, M. D. & Kreidenweis, S. M. A single parameter representation of hygroscopic growth and cloud condensation nucleus activity. *Atmospheric Chemistry and Physics* **7**, 1961-1971, doi:10.5194/acp-7-1961-2007 (2007).

25 Charlson, R. J., Lovelock, J. E., Andreae, M. O. & Warren, S. G. OCEANIC PHYTOPLANKTON, ATMOSPHERIC SULFUR, CLOUD ALBEDO AND CLIMATE. *Nature* **326**, 655-661, doi:10.1038/326655a0 (1987).

26 Ayers, G. P. & Gras, J. L. SEASONAL RELATIONSHIP BETWEEN CLOUD CONDENSATION NUCLEI AND AEROSOL METHANESULFONATE IN MARINE AIR. *Nature* **353**, 834-835, doi:10.1038/353834a0 (1991).

27 Hegg, D. A., Ferek, R. J., Hobbs, P. V. & Radke, L. F. DIMETHYL SULFIDE AND CLOUD CONDENSATION NUCLEUS CORRELATIONS IN THE NORTHEAST PACIFIC-OCEAN. *Journal of Geophysical Research-Atmospheres* **96**, 13189-13191, doi:10.1029/91jd01309 (1991).

28 Russell, L. M., Pandis, S. N. & Seinfeld, J. H. AEROSOL PRODUCTION AND GROWTH IN THE MARINE BOUNDARY-LAYER. *Journal of Geophysical Research-Atmospheres* **99**, 20989-21003, doi:10.1029/94jd01932 (1994).

29 Andreae, M. O., Elbert, W. & Demora, S. J. BIOGENIC SULFUR EMISSIONS AND AEROSOLS OVER THE TROPICAL SOUTH-ATLANTIC .3. ATMOSPHERIC DIMETHYLSULFIDE, AEROSOLS AND CLOUD CONDENSATION NUCLEI. *Journal of Geophysical Research-Atmospheres* **100**, 11335-11356, doi:10.1029/94jd02828 (1995).

30 Raes, F. ENTRAINMENT OF FREE TROPOSPHERIC AEROSOLS AS A REGULATING MECHANISM FOR CLOUD CONDENSATION NUCLEI IN THE REMOTE MARINE BOUNDARY-LAYER. *Journal of Geophysical Research-Atmospheres* **100**, 2893-2903, doi:10.1029/94jd02832 (1995).

31 Warren, D. R. & Seinfeld, J. H. PREDICTION OF AEROSOL CONCENTRATIONS RESULTING FROM A BURST OF NUCLEATION. *Journal of Colloid and Interface Science* **105**, 136-142, doi:10.1016/0021-9797(85)90356-x (1985).

32 Covert, D. S., Kapustin, V. N., Quinn, P. K. & Bates, T. S. NEW PARTICLE FORMATION IN THE MARINE BOUNDARY-LAYER. *Journal of Geophysical Research-Atmospheres* **97**, 20581-20589, doi:10.1029/92jd02074 (1992).

33 Pirjola, L., O'Dowd, C. D., Brooks, I. M. & Kulmala, M. Can new particle formation occur in the clean marine boundary layer? *Journal of Geophysical Research-Atmospheres* **105**, 26531-26546, doi:10.1029/2000jd900310 (2000).

34 Hegg, D. A., Radke, L. F. & Hobbs, P. V. PARTICLE-PRODUCTION ASSOCIATED WITH MARINE CLOUDS. *Journal of Geophysical Research-Atmospheres* **95**, 13917-13926, doi:10.1029/JD095iD09p13917 (1990).

35 Perry, K. D. & Hobbs, P. V. FURTHER EVIDENCE FOR PARTICLE NUCLEATION IN CLEAR-AIR ADJACENT TO MARINE CUMULUS CLOUDS. *Journal of Geophysical Research-Atmospheres* **99**, 22803-22818, doi:10.1029/94jd01926 (1994).

36 Clarke, A. D., Li, Z. & Litchy, M. Aerosol dynamics in the equatorial Pacific Marine boundary layer: Microphysics, diurnal cycles and entrainment. *Geophysical Research Letters* **23**, 733-736, doi:10.1029/96gl00778 (1996).

37 Raes, F., VanDingenen, R., Cuevas, E., VanVelthoven, P. F. J. & Prospero, J. M. Observations of aerosols in the free troposphere and marine boundary layer of the subtropical Northeast Atlantic: Discussion of processes determining their size distribution. *Journal of Geophysical Research-Atmospheres* **102**, 21315-21328, doi:10.1029/97jd01122 (1997).

38 Clarke, A. D. *et al.* Particle nucleation in the tropical boundary layer and its coupling to marine sulfur sources. *Science* **282**, 89-92, doi:10.1126/science.282.5386.89 (1998).

39 Katoshevski, D., Nenes, A. & Seinfeld, J. H. A study of processes that govern the maintenance of aerosols in the marine boundary layer. *Journal of Aerosol Science* **30**, 503-532, doi:10.1016/s0021-8502(98)00740-x (1999).

40 Reus, M. *et al.* Aerosol production and growth in the upper free troposphere. *Journal of Geophysical Research-Atmospheres* **105**, 24751-24762, doi:10.1029/2000jd900382 (2000).

41 Kazil, J., Lovejoy, E. R., Barth, M. C. & O'Brien, K. Aerosol nucleation over oceans and the role of galactic cosmic rays. *Atmospheric Chemistry and Physics* **6**, 4905-4924, doi:10.5194/acp-6-4905-2006 (2006).

42 Korhonen, H., Carslaw, K. S., Spracklen, D. V., Mann, G. W. & Woodhouse, M. T. Influence of oceanic dimethyl sulfide emissions on cloud condensation nuclei concentrations and seasonality over the remote Southern Hemisphere oceans: A global model study. *Journal of Geophysical Research-Atmospheres* **113**, doi:10.1029/2007jd009718 (2008).

43 Merikanto, J., Spracklen, D. V., Mann, G. W., Pickering, S. J. & Carslaw, K. S. Impact of nucleation on global CCN. *Atmospheric Chemistry and Physics* **9**, 8601-8616, doi:10.5194/acp-9-8601-2009 (2009).

44 Clarke, A. D. *et al.* Free troposphere as a major source of CCN for the equatorial pacific boundary layer: long-range transport and teleconnections. *Atmospheric Chemistry and Physics* **13**, 7511-7529, doi:10.5194/acp-13-7511-2013 (2013).

45 Ulbrich, I. M., Canagaratna, M. R., Zhang, Q., Worsnop, D. R. & Jimenez, J. L. Interpretation of organic components from Positive Matrix Factorization of aerosol mass spectrometric data. *Atmospheric Chemistry and Physics* **9**, 2891-2918, doi:10.5194/acp-9-2891-2009 (2009).

46 Crippa, M. *et al.* Identification of marine and continental aerosol sources in Paris using high resolution aerosol mass spectrometry. *Journal of Geophysical Research-Atmospheres* **118**, 1950-1963, doi:10.1002/jgrd.50151 (2013).

47 Liu, J. *et al.* Observational Evidence for Pollution-influenced Selective Uptake Contributing to Biogenic Secondary Organic Aerosols in the Southeastern US. doi:10.1002/2017gl074665 (2017).

48 Budisulistiorini, S. H. *et al.* Examining the effects of anthropogenic emissions on isoprene-derived secondary organic aerosol formation during the 2013 Southern Oxidant and Aerosol Study (SOAS) at the Look Rock, Tennessee ground site. *Atmospheric Chemistry and Physics* **15**, 8871-8888, doi:10.5194/acp-15-8871-2015 (2015).

49 Schmale, J. *et al.* Sub-Antarctic marine aerosol: dominant contributions from biogenic sources. *Atmospheric Chemistry and Physics* **13**, 8669-8694, doi:10.5194/acp-13-8669-2013 (2013).

50 Sanchez, K. J. *et al.* Meteorological and aerosol effects on marine cloud microphysical properties. *Journal of Geophysical Research-Atmospheres* **121**, 4142-4161, doi:10.1002/2015jd024595 (2016).

51 Chang, R. Y. W. *et al.* The hygroscopicity parameter (kappa) of ambient organic aerosol at a field site subject to biogenic and anthropogenic influences: relationship to degree of aerosol oxidation. *Atmospheric Chemistry and Physics* **10**, 5047-5064, doi:10.5194/acp-10-5047-2010 (2010).

52 DeCarlo, P. F., Slowik, J. G., Worsnop, D. R., Davidovits, P. & Jimenez, J. L. Particle morphology and density characterization by combined mobility and aerodynamic diameter measurements. Part 1: Theory. *Aerosol Science and Technology* **38**, 1185-1205, doi:10.1080/027868290903907 (2004).
